# Cotton pest and disease diagnosis via YOLOv11-based deep learning and knowledge graphs: a real-time voice-enabled edge solution

**DOI:** 10.3389/fpls.2025.1671755

**Published:** 2025-10-09

**Authors:** Meiqi Zhong, Linjing Wei, Henghui Mo

**Affiliations:** College of Information Science and Technology, Gansu Agricultural University, Lanzhou, Gansu, China

**Keywords:** cotton pest and disease detection, knowledge graph, voice interaction, model pruning, knowledge distillation

## Abstract

**Introduction:**

High labor costs, limited expert availability, and slow response hinder cotton pest and disease management. We propose a real-time, voice-enabled edge solution that integrates deep learning–based detection with a domain knowledge graph to deliver accessible, field-ready decision support.

**Methods:**

We construct a cotton pest–disease knowledge graph with over 3,000 triples spanning seven major categories by fusing expert-curated and web-sourced knowledge. For image recognition, we develop an enhanced YOLOv11 detector compressed via LAMP pruning and a teacher–assistant–student distillation strategy for lightweight, high-performance deployment on Jetson Xavier NX. Detected objects are semantically aligned to graph entities to generate context-aware recommendations, which are delivered through Bluetooth voice feedback for hands-free use.

**Results:**

The optimized model has 0.3M parameters and achieves ${\text{mAP}}_{50}$ = 0.835 at 52 FPS on the edge device, enabling stable real-time inference in field conditions while preserving detection accuracy.

**Discussion:**

Coupling a compact detector with a structured knowledge graph and voice interaction reduces dependence on expert labor and speeds response in non-expert settings, demonstrating a practical pathway to scalable, intelligent cotton pest and disease management at the edge.

## 1 Introduction

Cotton is recognized as one of the world’s most important economic crops, serving as a fundamental raw material for the textile industry and underpinning agricultural economies Wang and Memon (2020). Nevertheless, during the cultivation process, the occurrence of pests and diseases is frequent, which continues to pose major challenges to the sustainable development of the cotton industry Ahmad et al. (2020). In fact, it is estimated that annual losses in cotton yield due to such factors often surpass 20%, leading to substantial negative impacts on both farmers’ incomes and the overall health of the sector. Conventionally, the identification of cotton pests and diseases has relied on manual field surveys and the expertise of specialists. Although this approach can deliver reliable results in some cases, it is typically constrained by low efficiency, the limited availability of skilled personnel, and a non-negligible risk of misdiagnosis Buja et al. (2021). Compounding these issues are increasing labor costs and the ongoing outflow of rural populations, which have further intensified the need for advanced, automated detection technologies in modern agricultural production.

In recent years, rapid advances in artificial intelligence—especially deep learning—have substantially accelerated computer vision and opened new opportunities for automating the identification and management of crop pests and diseases LeCun et al. (2015). Among these, object detection has emerged as aprominent deep learning application, valued for its notable efficiency and accuracy. Well-established object detection models such as YOLO Jiang et al. (2022), Faster R-CNN Cheng et al. (2018), and SSD Liu et al. (2016a) have all shown strong performance in identifying pests and diseases across a range of crops. Still, while these algorithms are highly effective at classification, relying solely on image-based recognition is insufficient for providing thorough diagnostic reasoning or specific prevention recommendations. Bridging the gap between detection results and practical agricultural knowledge or actionable prevention measures continues to be a pressing challenge for the development of intelligent pest management systems.

Knowledge graph technology has recently gained ground in agriculture as an advanced means for representing and reasoning over domain knowledge Bhuyan et al. (2022). By integrating structured, semi-structured, and unstructured data sources, knowledge graphs offer an effective way to organize and visualize information relevant to pests and diseases, such as symptoms, transmission routes, pathogenic mechanisms, and recommended control measures. With the aid of reasoning methods, these graphs can support decision-making and problem-solving, and have already shown promising results in areas like healthcare, education, and agriculture Wang et al. (2017). That said, the construction of knowledge graphs focused on agricultural pests and diseases is still in its infancy. There is a clear need to further enhance their accuracy, scope, and usefulness in practical applications. Moreover, integrating knowledge graph resources with outputs from deep learning-based object detection, to realize a unified and intelligent system for diagnosis and prevention, is a direction that calls for ongoing research and innovation Zhu et al. (2023).

At the same time, advances in IoT and edge computing have steadily enhanced the computational capabilities of edge devices, making it increasingly practical to run complex deep learning models in real time Ai et al. (2018). Platforms such as NVIDIA’s Jetson Xavier NX stand out for their strong processing power, energy efficiency, and straightforward deployment, providing a solid technical foundation for implementing object detection and knowledge reasoning in agricultural environments. By integrating object detection with knowledge graph reasoning directly on edge devices, it becomes possible to achieve timely and accurate diagnosis of pest and disease issues, while also reducing network bandwidth usage and minimizing latency. These improvements contribute to more responsive and reliable decision-making in the field Chen et al. (2022).

In addition, many front-line agricultural practitioners have limited experience with information technology, which can make it challenging for them to interpret direct image recognition outputs or read written diagnostic reports. To address this, delivering diagnostic results and control suggestions to farmers via voice broadcasting helps to break down communication barriers Cole and Fernando (2021). This approach not only makes it easier for users to access key information but also supports greater convenience in field operations and promotes a higher degree of intelligence in agricultural production.

Building on the above background and considerations, this work presents an intelligent system for cotton pest and disease detection that brings together object detection, knowledge graph reasoning, and a voice interaction module to offer user-friendly results. The goal is to boost both the accuracy and efficiency of pest and disease diagnosis. In this study, an enhanced lightweight detection model for cotton diseases is developed and combined with knowledge graph methods to support decision-making. The main contributions are summarized as follows:

a. Introduces a multi-dimensional data augmentation approach utilizing StyleGAN-XL, which is leveraged to generate and diversify high-quality pest and disease images. This method effectively alleviates issues of limited and homogeneous datasets.
b. Applies the LAMP pruning technique for model compression and optimization, allowing the network to be efficiently deployed on edge devices like Jetson Xavier NX and thus meeting the practical requirements of field applications.
c. Proposes a novel teacher–assistant–student knowledge distillation framework, employing soft knowledge transfer to enhance the performance of the student model and offset accuracy reductions stemming from pruning.
d. Develops a cotton pest and disease knowledge graph by integrating crawled data, expert input, and knowledge fusion, with Neo4j-based graph storage and efficient reasoning to strengthen the system’s capabilities in diagnosis and prevention recommendation.

**Figure 1:**
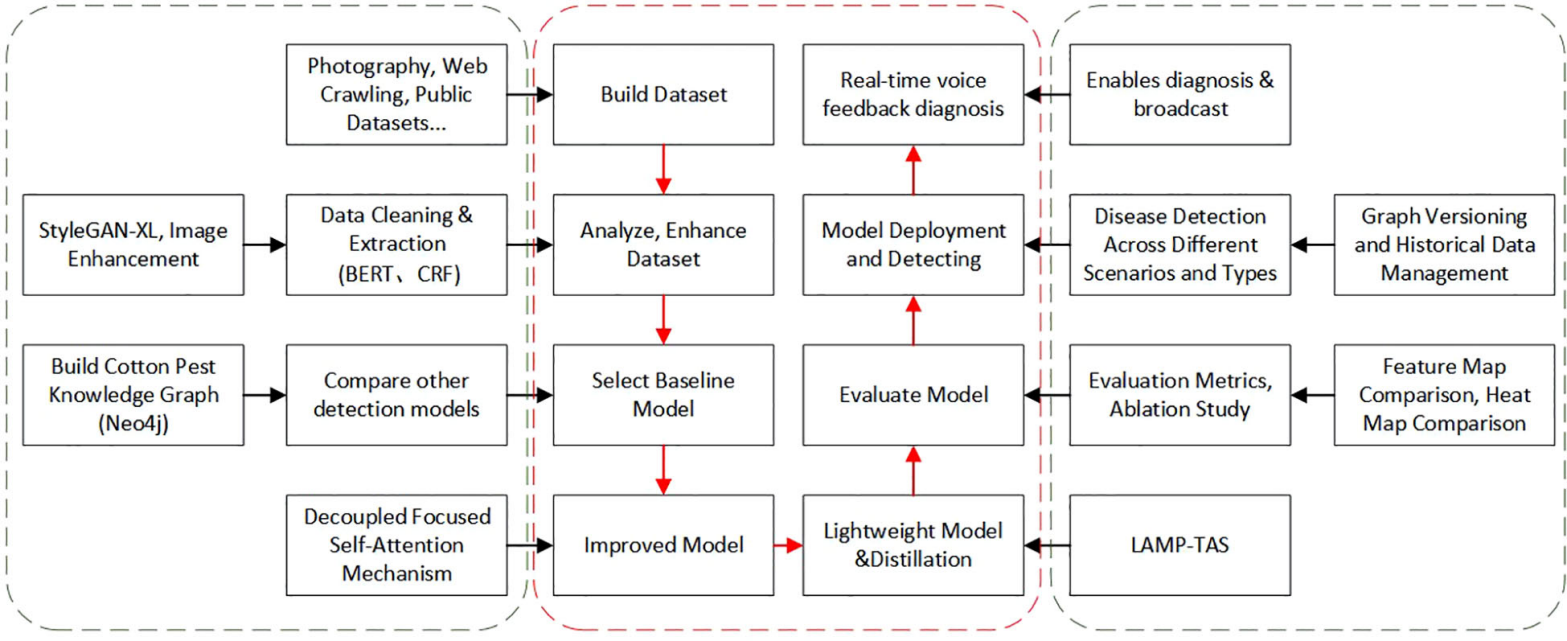
Main workflow diagram.

To illustrate the methods developed in this work, **Figure 1** summarizes the system architecture and the main modules, including image acquisition and preprocessing, knowledge graph construction for cotton pests and diseases, deep learning model optimization (pruning and distillation), edge deployment, and the voice interaction component. Section 2 reviews relevant literature. Section 3 outlines the system design and structure. Section 4 details the construction of the cotton pest and disease knowledge graph, covering data collection, entity extraction, graph storage, and reasoning. Section 5 describes the design and optimization of the YOLOv11-based object detection model, with a focus on data augmentation, LAMP pruning, and hierarchical knowledge distillation. Section 6 reports on experiments, model training settings, evaluation metrics, and performance analysis, including detection results and the Q&A system. Section 8 discusses current limitations and future improvements, while Section 9 concludes and suggests research directions.

## 2 Related work

Computer vision and knowledge graph technologies have shown considerable promise in agricultural applications. Initial efforts in pest and disease detection often relied on traditional image processing techniques, including color and texture feature analysis.

Ying et al. (2009) introduced median filters for image denoising, where the central pixel in a region is replaced by the median grayscale value of surrounding pixels. This technique was applied to image smoothing in color spaces such as CIELAB, YCbCr, and HSI, and has been used for detecting crop diseases in cotton, rice, and corn. Chaudhary et al. (2012) applied the Otsu method to determine optimal thresholds for preprocessed images in cotton leaf disease detection, demonstrating stable performance across various crops regardless of leaf type. Rathod et al. (2014) used median filters for denoising cotton leaf images in the CIELAB color space, then applied K-Medoid clustering to extract green leaf pixels for region of interest selection. They further calculated color and texture features and used neural networks for classification, reporting up to 96% accuracy with HS color features. Jenifa et al. (2019) combined median filtering and K-Means clustering to extract disease spots, using color and texture features to classify cotton leaf diseases. Multi-SVM models achieved 93.63% accuracy, outperforming CNNs by 7% and showing lower susceptibility to overfitting. El Sghair et al. (2017) employed median filtering and thresholding in the CIELAB color space to accurately extract disease areas, concluding that this color space is particularly suitable for identifying regions of interest. While traditional image processing approaches like median filtering, Otsu thresholding, and K-Means clustering can effectively reduce noise and extract lesions in specific color spaces, their performance is highly sensitive to lighting, background, and image quality. The inability to model complex nonlinear patterns often results in false positives or missed detections in cases with multiple coexisting diseases, small lesions, or overlapping symptoms. Limited generalizability and robustness restrict their suitability for large-scale field deployment, posing challenges for widespread adoption in agriculture.

In recent years, computer vision technologies—especially those based on deep learning—have found broad application in agricultural pest and disease detection, resulting in notable advances in performance. Convolutional Neural Networks (CNNs) have shown strong results in disease recognition and classification tasks. Jianhua et al. (2018) designed a cotton leaf disease detection system using an enhanced VGG network and data augmentation, leading to improved accuracy and generalization across disease types on standard datasets. Wang et al. (2018) used an adaptive discriminative deep belief network to boost cotton disease prediction, though real-world robustness remains a challenge. Muthukannan and Latha (2018) introduced a genetic algorithm-based feed-forward neural network (GA_FFNN) for identifying cotton and other plant diseases, achieving an average accuracy of 81.82%. Zhao et al. (2020) applied transfer learning to improve cotton disease and pest recognition, but observed reduced accuracy in complex scenes. Banu and Mani (2020) used pre-trained RESNET and VGG16 models for pathogen detection in cotton, with VGG16 and ResNet reaching 92.5% and 96.2% accuracy, respectively. Alves et al. (2020) developed a modified ResNet34 architecture for cotton disease identification, achieving 97.8% accuracy—outperforming methods like LBP-SVM, AlexNet, ResNet34, and ResNet50. Vishnoi et al. (2022) proposed a CNN-based approach for apple leaf disease detection that uses data augmentation and a shallow network to lower computation and storage needs. The model achieved 98% accuracy on the PlantVillage dataset and is suitable for deployment on resource-limited devices like handhelds. These strategies have significantly improved classification performance and generalization. Still, while deep learning models raise disease recognition accuracy, challenges remain in ensuring robustness under real field conditions, and high computational demand limits their use on edge devices. Traditional deep learning models also show clear shortcomings in generalization and classification in diverse scenarios Zhang et al. (2021).

With the advancement of object detection technology, one-stage detectors—particularly the YOLO series—have seen widespread use in agricultural applications due to their fast real-time inference. Zhang et al. (2022) introduced a real-time high-performance model based on improved YOLOX, which achieved 94.60% average precision in cotton disease detection by incorporating Efficient Channel Attention (ECA) and Focal Loss. Susa et al. (2022) applied YOLOv3 for real-time disease detection in tea gardens, obtaining a mean average precision (mAP) of 86%. Zhang et al. (2024b) developed YOLO SSPD—a small-target cotton boll detection model based on YOLOv8—that used spatial-to-depth convolution, non-stride convolution, and a parameter-free attention mechanism to boost detection accuracy for small objects. The model reached 87.4% accuracy on drone imagery and showed strong performance in boll counting. Zhang et al. (2025) proposed LMS-YOLO11n, which improved ${\text{mAP}}_{50}$ by 2.5% in cotton weed detection through multi-scale feature fusion and structural optimization, reducing parameters by 37% and increasing speed. Lee et al. (2025) optimized hyperparameters for YOLOv11m in tomato leaf disease recognition using OFAT and random search, resulting in the C47 model with $xtmAP_{50}$ of 0.99262, and precision and recall rates above 99%, confirming its practical value in crop disease recognition. Collectively, these studies highlight the potential of object detection to enhance real-time agricultural pest and disease monitoring. However, existing approaches still require further improvement for small object detection and complex scene performance.

Meanwhile, knowledge graph technology has gained increasing attention in the agricultural domain. Through structured representation and reasoning, knowledge graphs can support agricultural decision-making and diagnosis. Bhuyan et al. (2024) introduced UrbanAgriKG, a knowledge graph for urban agriculture that covers entities and relationships such as farms, crops, technologies, and environmental factors. Their study also compared graph embedding techniques for link prediction and similarity search, highlighting the value of combining knowledge graphs with representation learning for urban agriculture. Furqon and Bukhori (2023) built a knowledge graph to organize expert knowledge about rice pests and diseases in Indonesia, using SPARQL extraction to populate pest and disease instances, forming a connected, structured knowledge base that aids rice disease management. Choudhary et al. (2020) integrated domain ontologies, sensor data, and weather data into a knowledge graph, connecting multi-source agricultural information (soil, climate, crop types) and aggregating it for machine learning models via SPARQL, demonstrating high practicality. Ge et al. (2024) combined expert knowledge graphs and case-based reasoning for intelligent rice fertilization recommendations. Their system used a semantic knowledge base—covering soil, growth stages, nutrients, etc.—and matched historical cases for tailored fertilizer advice, showing high experimental accuracy. Jing and Li (2024) also constructed a heterogeneous agricultural product knowledge graph spanning production to food processing, linking products to attributes such as pesticide residues and storage. It proved effective for traceability and quality control in tests with 100 carrot batches. Zou et al. (2023) created a county-level corn ecological knowledge graph (including climate and soil data) and used RippleNet for recommending planting regions, achieving 76.3% accuracy across 331 varieties and 59 sites, outperforming various machine learning and graph neural network baselines. Alharbi et al. (2024) developed a dual-ontology system for pest and disease knowledge and explainable diagnostics, enabling farmers to input symptoms and receive matched diagnoses, causal factors, and literature-based explanations, increasing system credibility. Jiang et al. (2025) proposed a two-stage fusion approach for integrating multiple pig disease databases, aligning entities and using multi-view enhancement. The resulting knowledge graph supports veterinary diagnostics and planning by covering diseases, symptoms, treatments, and epidemiology, providing a comprehensive foundation for the pig industry. Despite these advances, most agricultural knowledge graph research remains theoretical, with limited integration into practical systems like object detection for real-world deployment.

Alongside progress in computer vision and knowledge representation, recent smart agriculture research has increasingly focused on integrating Internet of Things (IoT) and machine learning technologies to improve real-time sensing and decision-making. For instance, Khan et al. (2022a) developed an IoT-based, context-aware fertilizer recommendation platform that combines real-time soil fertility maps with machine learning, supporting more accurate and efficient fertilization. In irrigation management and saline soil improvement, Khan et al. (2022b) proposed a context-aware evapotranspiration estimation system using IoT sensors and ensembled LSTM models, which achieved strong performance in real-world field applications. Additionally, Bashir et al. (2023) presented an ensemble artificial neural network method for optimizing reference evapotranspiration calculations, offering flexibility in parameter selection and high accuracy for precision irrigation at scale. Collectively, these studies reflect a shift toward data-driven, context-aware, multi-source sensing strategies in smart agriculture, complementing advances in computer vision and knowledge graph reasoning.

Recent studies have started to explore integrating knowledge graphs with deep learning. Li et al. (2024) introduced a deep model that combines Diffusion Transformer with knowledge graphs for efficient detection of cucumber leaf diseases. By embedding crop disease knowledge into the Transformer’s attention mechanism, the approach addresses complex features and class imbalance, achieving 93% precision, 89% recall, 91% mAP, and a real-time speed of 57 FPS. The model was also compressed for mobile field use. Gao et al. (2024) proposed a cotton pest and disease detection system that fuses visual Transformer networks with knowledge graphs and deploys on edge devices. Knowledge graphs add agricultural context for improved feature learning, and the Transformer backbone enhances robustness. On mobile platforms, their model reached 94% detection accuracy and 0.95 mAP, surpassing YOLOv8 and RetinaNet by 3–14%, with speeds around 49.7 FPS. Chhetri et al. (2023) combined deep neural networks and semantic knowledge graphs for cassava leaf disease recognition, improving accuracy to 90.5% and providing explainable outputs, with 95% of users finding the explanations helpful. The method improves on the interpretability and context limitations of conventional deep models. Wang et al. (2024) developed a tomato pest and disease knowledge graph using deep NER models (ALBERT-BiLSTM-CRF) to extract entities from agricultural texts, achieving a 95.03% recall rate after integrating multi-source datasets. The resulting knowledge, stored in Neo4j, supports digital tomato disease diagnosis. Lv et al. (2024) proposed Veg-MMKG, a multimodal vegetable knowledge graph method that fuses text and images, using pre-trained models and dual-stream learning for text-image alignment. Their approach employs cross-modal contrastive learning, reaching 76.7% accuracy in image-text agricultural queries. Yan et al. (2025) developed CropDP-KG, a large-scale pest and disease knowledge graph for Chinese crops, extracting over 13,000 entities and 21,000 relationships from national datasets to unify data standards. Their system and data are open-source, aiding precision agriculture. Chen et al. (2019) released AgriKG, a large agricultural graph capturing knowledge on crops, pests, soils, climate, and management via NLP and deep learning, and providing support for agricultural QA and entity search. Although these integrations offer new pathways for practical deployment, most work is still at the exploratory stage and lacks validated, systematic solutions for real-world agricultural environments Shahin et al. (2017).

To advance smart agriculture in practical settings, this work introduces an intelligent cotton pest and disease detection system that brings together object detection, knowledge graph reasoning, and voice interaction. Built on the Jetson Xavier NX platform, the system adopts a lightweight model and fast response design, supporting real-time field identification. By developing a dedicated knowledge graph and integrating voice broadcast, the system closes the loop from recognition to reasoning to user feedback. This approach reduces operational barriers for farmers, improves the practicality and intelligence of agricultural AI, and offers a viable path toward intelligent agricultural development.

## 3 System overall design and framework

### 3.1 System design objectives and overall architecture

**Figure 2:**
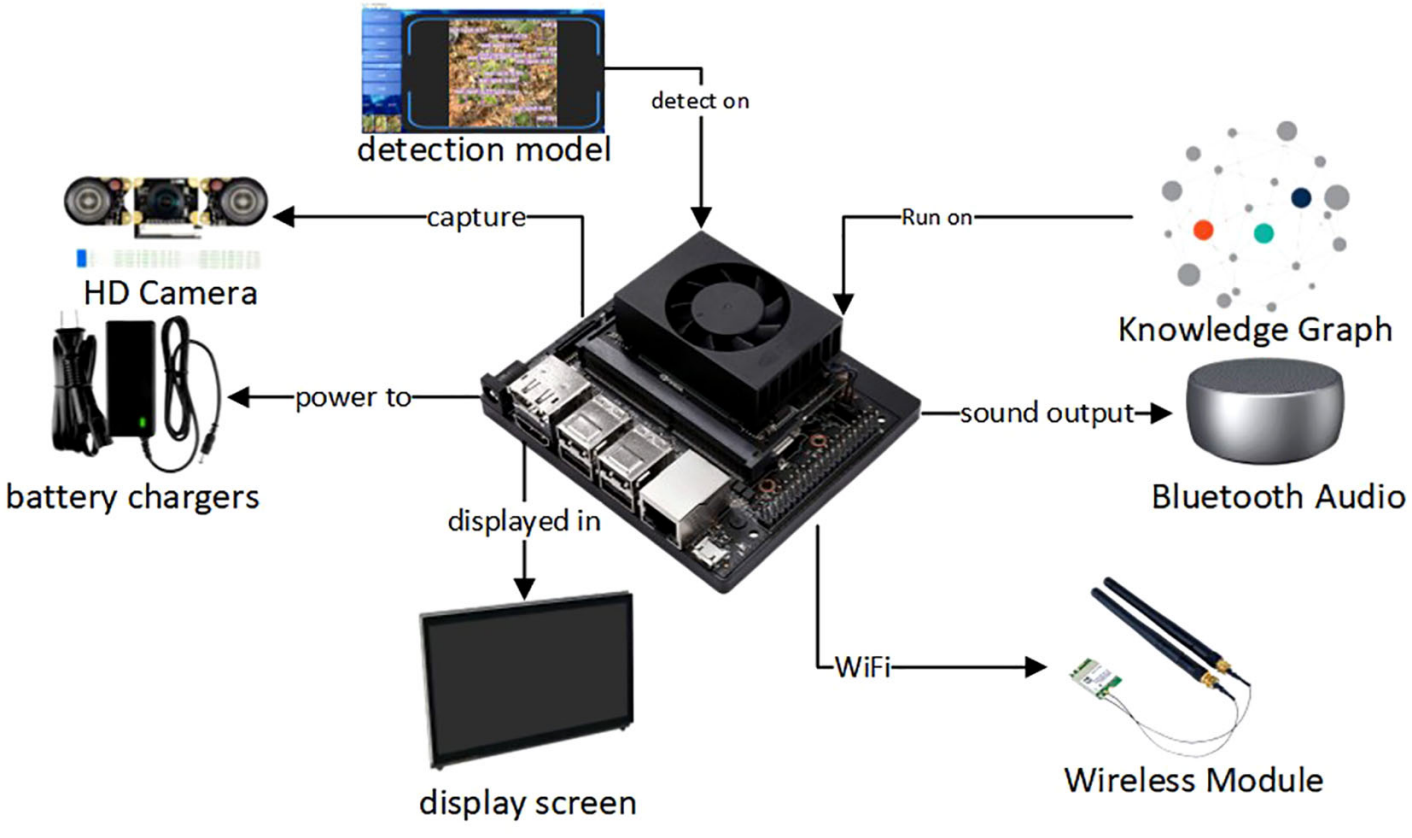
Overview of the system architecture.

The goal of this system is to deliver an intelligent recognition and interaction platform suitable for real-world agricultural use. By combining image analysis, knowledge-based reasoning, and voice output, it supports a closed-loop diagnostic process from perception to explanation. The overall architecture is outlined in **Figure 2**. In its design, the system balances high accuracy and speed with structured management of semantic data and clear, user-friendly outputs, aiming to give farmers reliable and accessible decision support.

### 3.2 Image acquisition and preprocessing module

The image acquisition module acts as the system’s primary sensing interface. Field cameras with autofocus and adaptive lighting ensure clear imaging in various weather and lighting conditions. Acquired images are processed with exposure correction, gamma adjustment, grayscale normalization, and edge enhancement to reduce noise from shadows and complex backgrounds. To further improve robustness against varied angles and occlusions, the module employs multi-angle supplementary capture and uses slight camera shifts for redundant image fusion.

### 3.3 Object detection module

The object detection module locates and classifies pest and disease regions in the input images, using a YOLOv11n-based model designed for fine-grained lesion detection on leaf surfaces. To lower deployment costs, the model is pruned with LAMP for lightweight operation, then converted to ONNX and accelerated with TensorRT for efficient inference on Jetson Xavier NX. This setup delivers millisecond-level detection speed. The module also includes confidence scoring and multi-target reordering, supporting prioritized, multi-label output when multiple diseases are present.

### 3.4 Knowledge graph reasoning module

The knowledge graph module, developed on the Neo4j platform, captures the full lifecycle of major cotton pests and diseases—covering pathogens, symptoms, transmission, climate triggers, prevention strategies, and related semantic links Huang and Dong (2013). Hierarchical reasoning and disease comparison are supported using weighted relationship chains and path rules, and the system can recommend entities even when input labels are unclear. To support scalability, expert knowledge can be added incrementally, and an entity disambiguation mechanism is included to reduce errors from label ambiguity Zhang et al. (2024a). The resulting structured knowledge is formatted for natural language output in the voice module.

### 3.5 Edge computing inference fusion module

This module underpins the system’s intelligence and autonomy, with all models and knowledge graphs running locally on the Jetson Xavier NX to support offline use. Thread pool management allows parallel execution of detection and reasoning, making full use of edge computing resources. A cache-first strategy is used for knowledge queries: common disease data and recommendations are stored in local memory for rapid, second-level responses when labels match. For field deployment, the system features low-power operation and battery management, supporting over six hours of continuous use without external power.

### 3.6 Voice interaction module

The voice interaction module delivers structured diagnoses and advice as natural language speech using a lightweight TTS engine, with support for multiple languages, adjustable speed, and keyword emphasis Li et al. (2021). It features intelligent broadcast control, Bluetooth output, and interruption handling to avoid repeated messages and ensure clear, timely feedback even when screens are unavailable. For ambiguous or multi-label results, all findings and suggestions are read out in turn; if uncertainty persists, the system requests clarification or summarizes possible scenarios for the user.

### 3.7 Module integration and system interaction mechanism

RESTful APIs manage communication between system modules, with all data exchanged in structured JSON format. Once the object detection module outputs pest and disease labels, results are sent via middleware to the knowledge graph engine. The engine returns structured explanations, which are then formatted with language templates for voice output, creating a full loop from detection to broadcast. All modules operate asynchronously and concurrently, reducing transmission delays and improving user response times.

### 3.8 Deployment adaptability and environmental robustness

The system is designed with practical agricultural deployment in mind: cameras are IP65-rated for water resistance, the main control unit is protected against overheating and shock, and the voice module features outdoor noise reduction. To handle power and network disruptions, the system includes auto-recovery, cache sync, and an offline knowledge mode. Image quality is monitored in real time using contrast and clarity metrics, with automatic re-capture triggered if images are blurred or blocked. Offline operation is supported, with daily data sync when a connection is available. During offline periods, detection results and logs are saved locally, and a differential sync protocol uploads only new or changed records when reconnected, minimizing bandwidth use. The sync supports resumable transfers with automatic retries. A dedicated battery and power management enable over six hours of autonomous operation, including safe shutdown and recovery. Transaction logs and checkpoints protect data integrity, ensuring the system remains reliable under field power and bandwidth constraints.

## 4 Construction of cotton pest and disease knowledge graph

### 4.1 Objectives and methods of knowledge graph construction

Knowledge graphs play a crucial role as the bridge connecting data and knowledge layers, systematically bringing together fragmented and heterogeneous agricultural information. This integration supports effective diagnosis and decision-making for cotton pest and disease prevention. In agricultural contexts, pest and disease problems typically involve complex, multi-dimensional relationships, making it difficult for traditional databases and search methods to deliver deep reasoning support. The cotton pest and disease knowledge graph developed in this study follows the design principles of comprehensive information integration, efficient semantic reasoning, and in-depth decision support. Its purpose is to provide richer, more accurate semantic details and targeted prevention advice following the initial identification of diseases by object detection.

In this study, the knowledge graph brings together data from various sources, including agricultural publications, expert repositories, online encyclopedias, and government documents. Through this integration, a semantic network is established that covers six main entities: disease categories, symptom profiles, pathogen types, stages of infection, transmission pathways, and prevention strategies Chen et al. (2024). This network structure supports dynamic reasoning and fast retrieval by capturing semantic links among different entities.

In terms of structural design, the graph adopts a directed graph representation method, as defined in (Equation 1):


(1) G=(V,E,R)

Where *V* represents the entity collection, *E* represents the relationship edge collection between entities, and *R* represents the relationship type collection. Each entity node is mapped to high-dimensional space to form vector representations for subsequent entity semantic similarity calculations *h* ∈ *R^d^*. Graph embedding is implemented through the TransE algorithm, and the optimization objective is defined in Equation (2):


(2) $$\;L=\sum_{(h,r,t)\in E}\sum_{(h^{\prime },r^{\prime },t^{\prime })\notin E}\text{max}(0,\gamma +d(h+r,t)-d(h^{\prime }+r,t^{\prime }))$$

where h, r, t respectively represent the head entity, relation and tail entity, d represents the distance between entities, *γ* represents the boundary threshold, and max(0,·) represents the positive and negative pair distinction in the form of Hinge Loss.

### 4.2 Data acquisition and information processing methods

During the data acquisition phase, this study collected publicly available online resources using custom web crawlers, sourcing data from platforms such as Baidu Encyclopedia and the Ministry of Agriculture and Rural Affairs pest and disease database. To process unstructured text, tools like HanLP and BERT-CRF were applied for named entity recognition and relationship extraction. Field technicians were also involved to annotate and verify terminology, ensuring the reliability and domain accuracy of the dataset. For knowledge fusion, entities were aligned using semantic vector space modeling. Contextual embeddings for candidate entities were generated with the pre-trained BERT model, followed by calculation of cosine similarity to assess the relationships between entities:


(3) $$\text{sim}(A,B)=\frac{A\cdot B}{\left\|A\|\cdot \|B\right\|}$$

Where A and B are the vector representations of the candidate entities. When sim(*A,B*) *>* 0.85, they are considered the same entity. Additionally, we designed a manual expert verification mechanism to handle edge cases that the model cannot accurately judge completely, thereby improving the quality of the knowledge graph.

To provide a clear visualization of the entity coverage and core conceptual structure of the knowledge graph developed in this research, **Table 1** summarizes examples of seven major entity classes represented in the system. These categories form the essential node types in the semantic network of the graph and correspond to the principal knowledge domains relevant for pest and disease identification and management in agricultural contexts. Representative examples are presented for each class, including key cotton pest and disease types (such as aphids, armyworms, leaf spot, etc.), along with their associated transmission and control information, which are the primary focus of the system.

**Table 1:** Examples of core entities in the knowledge graph.

Entity type	Examples
Diseases	Leaf spot, Bacterial blight, Fusarium wilt, Grey mildew, Leaf curl
Pests	Aphids, Armyworm
Symptoms	Yellowing leaves, Brown lesions, Leaf margin curling, Rot, Shedding, etc.
Pathogens	Fusarium spp., Botrytis cinerea, etc.
Infection stages	Seedling stage, Flowering and boll stage, Blooming stage
Transmission modes	Soil-borne, Airborne, Vector-borne
Control methods	Chemical spraying, Bio-agents, Physical barriers, Crop rotation, etc.

#### 4.2.1 Schema and worked example for Neo4j integration

Schema mapping. We map the seven detection targets to two top-level Neo4j labels::Pest (Aphids, Armyworm) and:Disease (Leaf spot, Leaf blight, Fusarium wilt, Grey mold, Leaf curl). These are linked to five auxiliary domains for diagnosis and recommendation:Pathogen,:Symptom,:TransmissionMode,:GrowthStage, and:ControlMethod. **Table 2** summarizes the main relation types and representative node attributes used by the reasoning module.

**Table 2:** Core relation types and representative node attributes in the Neo4j graph.

Relation	Pattern	Key attributes (node-level)
HAS_SYMPTOM	(:Disease)-[:HAS_SYMPTOM]->(:Symptom)	Symptom.name, region
CAUSED_BY	(:Disease)-[:CAUSED_BY]->(:Pathogen)	Pathogen.name, genus
TRANSMITTED_BY	(:Disease)-[:TRANSMITTED_BY]->(:TransmissionMode)	TransmissionMode.type
AFFECTS_STAGE	(:Disease)-[:AFFECTS_STAGE]->(:GrowthStage)	GrowthStage.name
TREATED_BY	(:Disease|:Pest)-[:TREATED_BY]->(:ControlMethod)	ControlMethod.type, intervaldays, dosagegL

Worked example: “Leaf spot”. Listing 1 instantiates a minimal actionable subgraph that connects:Disease{name:”Leaf spot”} to representative pathogens, symptoms, and control methods. We use MERGE with stable identifiers (id) to keep updates idempotent.

# Listing 1: Instantiation (MERGE) of a Leaf spot subgraph

MERGE (d:Disease {id:’dis_leaf_spot’, name:’Leaf spot’})

MERGE (p:Pathogen {id:’pat_alt_spp’, name:’Alternaria spp.’, genus:’Alternaria’})

MERGE (s1:Symptom {id:’sym_brown_circ’, name:’brown circular lesions’})

MERGE (s2:Symptom {id:’sym_yellow_halo’, name:’yellow halo around spots’})

MERGE (tm:TransmissionMode {id:’tm_airborne’, type:’airborne’})

MERGE (gs:GrowthStage {id:’gs_boll’, name:’boll stage’})

MERGE (cm1:ControlMethod {id:’cm_bio_bs’,

type:’bio-agent’, product:’Bacillus subtilis’,

interval_days:7, dosage_g_L:1.0})

MERGE (cm2:ControlMethod {id:’cm_chem_polyoxin’,

type:’chemical’, product:’polyoxin’,

interval_days:7, dosage_g_L:0.6})

MERGE (d)-[:CAUSED_BY]->(p)

MERGE (d)-[:HAS_SYMPTOM]->(s1)

MERGE (d)-[:HAS_SYMPTOM]->(s2)

MERGE (d)-[:TRANSMITTED_BY]->(tm)

MERGE (d)-[:AFFECTS_STAGE]->(gs)

MERGE (d)-[:TREATED_BY]->(cm1)

MERGE (d)-[:TREATED_BY]->(cm2);

*Note.* The example shows representative connections; in practice, multiple:Pathogen and:ControlMethod nodes can be attached with provenance tags from curated sources.

Actionable retrieval. For recommendation and voice output, we expand a disease-centered, 2-hop neighborhood and return structured fields (Listing 2):

# Listing 2: Retrieval of an actionable neighborhood for “Leaf spot”

MATCH (d:Disease {name:’Leaf spot’})

OPTIONAL MATCH (d)-[:CAUSED_BY]->(p:Pathogen)

OPTIONAL MATCH (d)-[:HAS_SYMPTOM]->(s:Symptom)

OPTIONAL MATCH (d)-[:TREATED_BY]->(cm:ControlMethod)

OPTIONAL MATCH (d)-[:TRANSMITTED_BY]->(tm:TransmissionMode)

OPTIONAL MATCH (d)-[:AFFECTS_STAGE]->(gs:GrowthStage)

RETURN d.name AS disease,

collect(DISTINCT p.name) AS pathogens,

collect(DISTINCT s.name) AS symptoms,

collect(DISTINCT {type:cm.type, product:cm.product,

interval:cm.interval_days, dosage:cm.dosage_g_L}) AS controls,

collect(DISTINCT tm.type) AS transmission,

collect(DISTINCT gs.name) AS stages;

Disambiguation and fusion. When aligning text-derived entities to the graph, we combine (i) BERT-based cosine similarity (threshold 0.85; see Equation 3) for semantic proximity, (ii) a curated gazetteer for high-precision matches of scientific/common names, and (iii) an expert-overrides rule for edge cases. Conflicts are resolved by priority: *expert > gazetteer > model score*. This keeps fusion deterministic while allowing incremental expert curation.

### 4.3 Integration of graph storage, reasoning, and maintenance mechanisms

**Figure 3:**
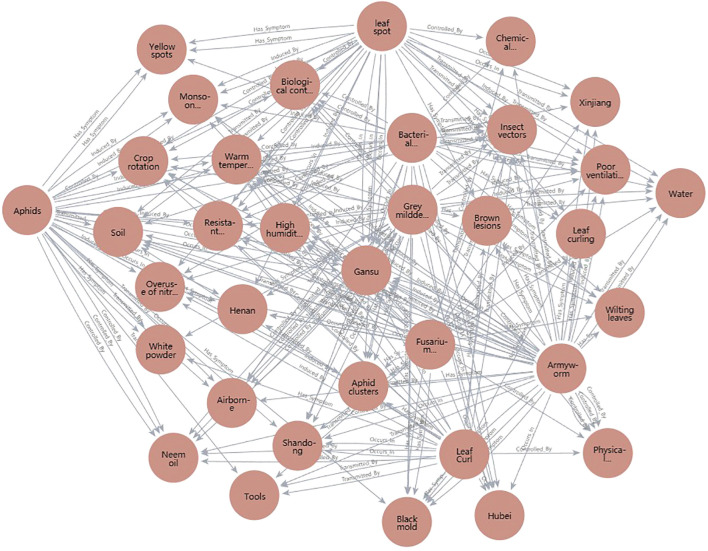
Schematic diagram of cotton pest and disease knowledge graph structure (entity-relationship view).

**Figure 4:**
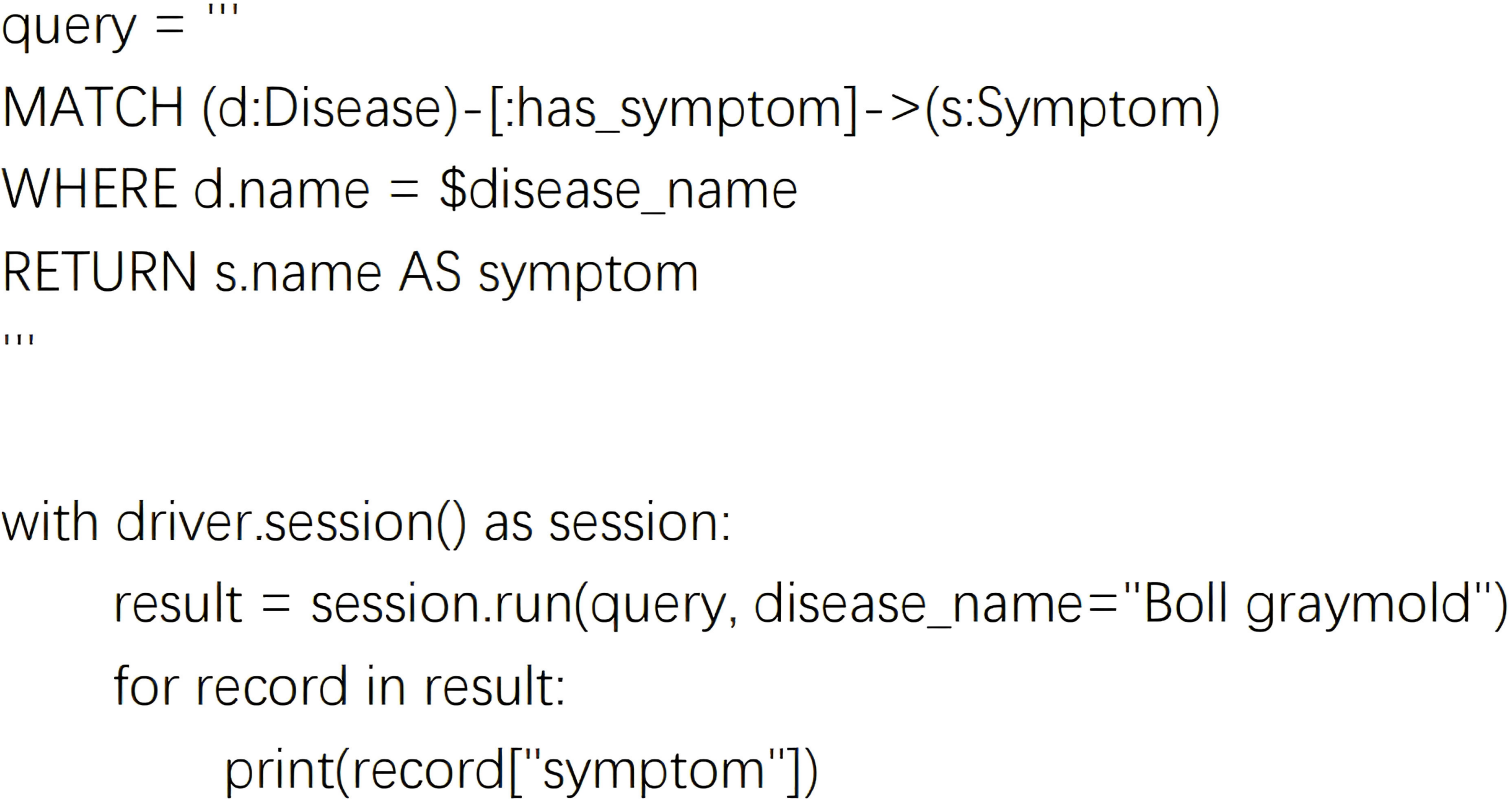
Semantic path matching for querying disease-related symptoms in the knowledge graph.

To enable effective semantic responses and strategy recommendations after recognition, the knowledge graph’s query speed, reasoning depth, and update flexibility are fundamental to maintaining usability and continuous system intelligence. The knowledge graph is stored using Neo4j, where entities and relationships are represented as nodes and directed edges, supporting efficient graph traversal (**Figure 3**). With Python interfaces to Neo4j, the system supports querying, inserting, and updating any entity or path, leveraging Cypher queries integrated with Python scripts to meet deployment needs. As illustrated in **Figure 4**, sample code can retrieve all symptom nodes related to “Cotton Gray Mold” within the graph.

This query retrieves all symptom nodes related to cotton gray mold and can be similarly extended to search for medicinal controls or transmission routes. To enhance response efficiency, we implemented a high-priority index system for frequently accessed entities and added a cache management module based on historical queries, allowing the graph to remain lightweight even under high concurrency and during edge-side inference. For reasoning, the system utilizes a configurable engine built on semantic path templates. For instance, when “leaf blight” is detected, the following reasoning chain may be constructed: “Leaf blight” → “Rice blast fungus” (caused by) → “Air transmission” (transmitted by) → “High humidity above 25°C” → “Biological agents + drug rotation” (treated by). These reasoning steps are dynamically updated based on environmental conditions and rules, producing natural language summaries such as: “Detected as cotton leaf blight, which is prevalent during the boll stage under humid conditions. Recommend applying Bacillus subtilis and polyoxin in rotation, spraying every 7 days.” This output is passed to the voice module for real-time user feedback. For graph updates, a hybrid mechanism combines automatic crawling and extraction with expert manual validation. Each month, the system collects new agricultural knowledge from trusted sources. New entities and relationships are extracted by NLP models, after which plant protection experts review, resolve conflicts, and remove outdated or erroneous data. This two-step process ensures update reliability by merging frequent automatic updates with periodic expert checks. At present, the knowledge graph is refreshed monthly; future improvements will focus on real-time incremental learning, so new knowledge—detected in the field or input by experts—can be integrated immediately. Nevertheless, challenges such as expert availability and resource constraints on edge devices must be considered for real-time deployment. The system is designed to continually integrate new information from real-time detection, allowing both the knowledge graph and detection model to be updated iteratively. This two-way update approach not only improves recognition accuracy, but also keeps the knowledge base aligned with current agricultural developments. Looking ahead, future work will focus on further automating real-time incremental updates, aiming to enhance the system’s adaptability to changing and complex field conditions.

**Figure 5:**
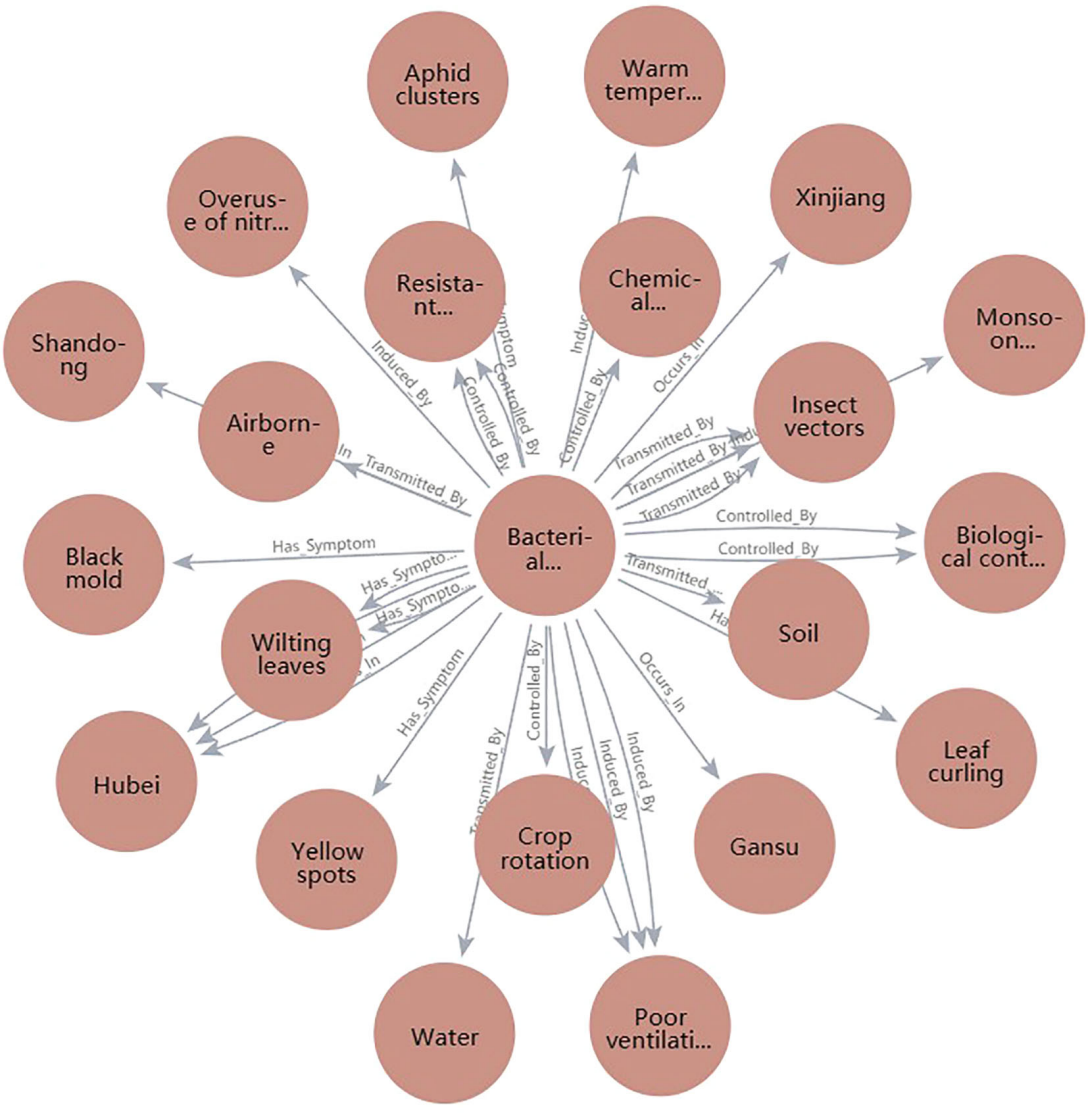
Instance diagram of leaf blight graph reasoning path (including recommended strategy paths).

**Figure 5** further illustrates ‘leaf blight’ as the core node, demonstrating the multi-hop path nodes activated during reasoning and the result fusion mechanism.

With the integration of these modules, the knowledge graph has progressed from a passive query tool to a dynamic, semantically-driven core component. Its ability to support the full workflow—from detection and recognition through to decision broadcasting—has paved the way for practical agricultural cognitive intelligence solutions.

## 5 Deep learning based object detection model

### 5.1 Dataset construction and distribution design

In deep learning-based pest and disease detection, datasets serve not only as the basis for model training, but also as critical determinants of generalization, robustness, and deployment performance. In this study, a high-quality image dataset for cotton leaf disease and pest recognition was built to reflect real-world conditions, with a focus on incorporating “scene complexity” and “semantic diversity” into the early stages of model development. This approach provides a strong foundation for ensuring both the transferability and practical effectiveness of the detection models in real deployments.

The dataset comprises 8,000 images representing seven primary cotton pest and disease categories: aphids, armyworms, leaf spot, leaf blight, Fusarium wilt, gray mold, and leaf curl, along with healthy samples to aid category differentiation and improve boundary feature learning. Data collection covered major cotton-producing regions, including southern Xinjiang, the central Yellow River Basin, and central Hubei high-yield zones. Sampling times ranged from sunny and post-rain conditions to cloudy weather and dawn/dusk, aiming to build a “cross-scene, cross-time, cross-region” semantic space that captures the diverse visual presentations of pests and diseases across different environmental and temporal settings. For image acquisition, both handheld and low-angle ground methods were employed. Ground-level images facilitated detailed capture of lesion patterns, while drone-based images contributed scale awareness and spatial context of the fields. Multi-angle approaches—including overhead, upward, and oblique perspectives—were systematically used to enhance the dataset’s robustness to viewing angles, reducing model reliance on specific camera positions.

In addition, supplementary data was obtained through web crawling and open-source image repositories. An automated crawler framework was implemented using keyword matching and image filtering to collect relevant pest and disease imagery from various agricultural technology platforms and open-access image sources. The crawler system featured a distributed, multi-threaded architecture, integrating image hash based deduplication and automated label classification to preliminarily organize and filter collected images. To ensure both image quality and annotation reliability, three plant protection experts were engaged to manually review and verify the labels of filtered samples, with particular emphasis on challenging categories such as “early-stage gray mold,” “late-stage armyworm aggregation,” and “leaf blight under backlighting”—cases that are rare but crucial in original field photography.

**Figure 6:**
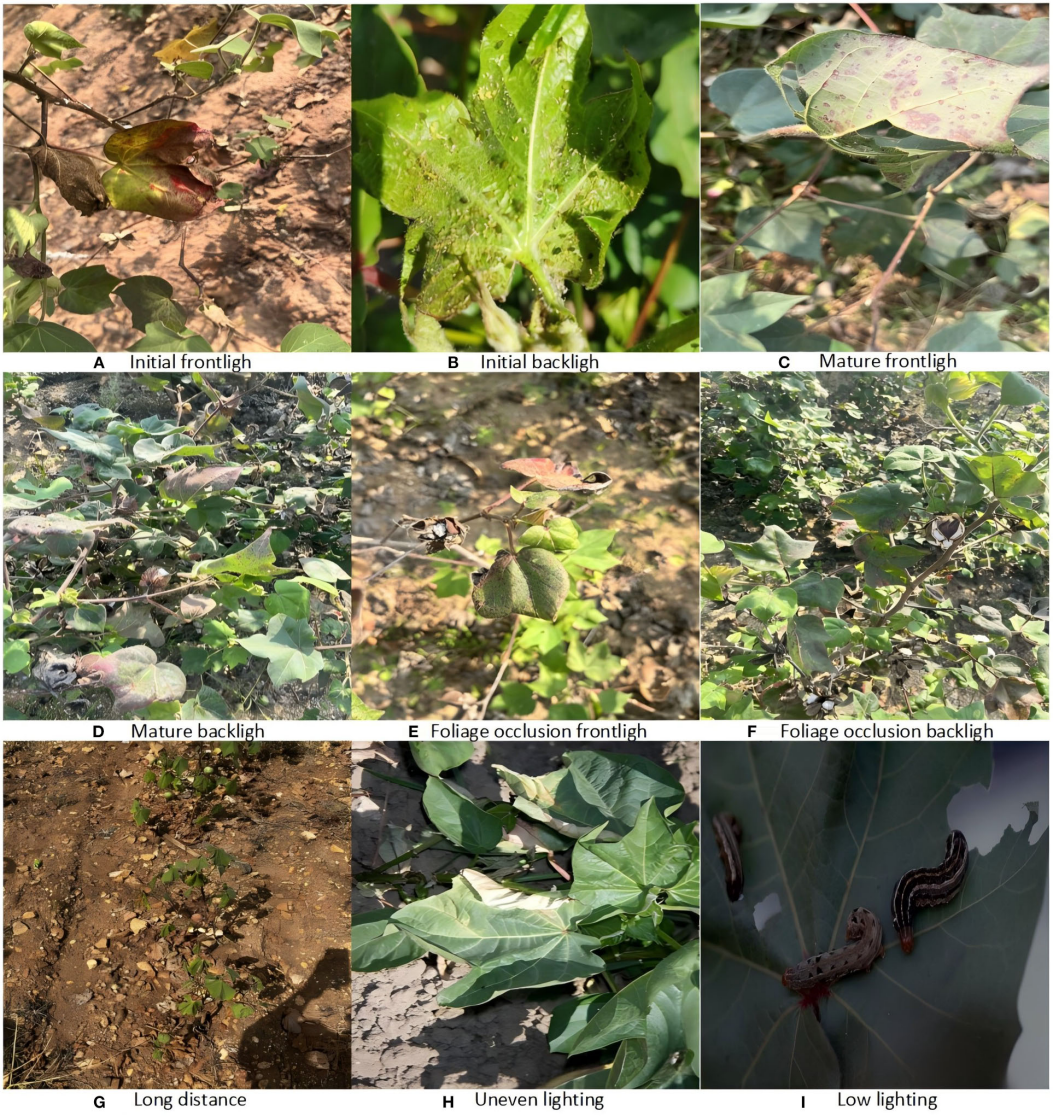
Dataset legend in complex environments.

All images were standardized to a resolution of 640×640 or higher to retain essential micro-level details, including leaf vein patterns, insect contours, and lesion edge diffusion. Additionally, diverse sources of interference—such as backlighting, variable illumination, branch and leaf occlusion, and water stain artifacts—were intentionally included to improve the model’s robustness in non-ideal conditions, as illustrated in **Figure 6**.

#### 5.1.1 StyleGAN-XL training method

To tackle the challenge of insufficient data for certain cotton pest and disease categories and scenarios, this study introduced an image synthesis mechanism based on StyleGAN-XL to generate high-quality, semantically diverse synthetic samples, thereby improving the detection model’s performance in few-shot and complex cases Melnik et al. (2024). StyleGAN-XL, known for its high resolution and controllability, excels at reproducing structural details and texture variations—making it ideal for simulating high-frequency features and color patterns in agricultural lesion imagery. For this work, StyleGAN-XL was trained using NVIDIA’s distributed multi-GPU framework, with both original and web-sourced images resized to 256×256 as input. The Generator features a separated latent space with dual-space mapping, projecting a Gaussian latent vector $z\in {\mathbb{R}}^{64}$ to a style space $w\in {\mathbb{R}}^{512}$ , and employs 14 synthesis layers with up to 1,024 channels to accurately capture fine lesion characteristics. To enhance spatial coherence and semantic variety, alias-free convolution and style mixing (with a 0.9 probability) are utilized. The Discriminator employs a Projected Discriminator architecture incorporating DeiT-Base Transformer and EfficientNet Lite0 as backbones, along with multi-scale branches and Differentiable Augmentation, enabling robust spatial-semantic fusion and effective handling of scale diversity in disease images. The generator and discriminator losses are given in Equations (4) and (5).


(4) $$L_{G}=E_{z∼N(0,1)}[-D(G(z))+\lambda_{\text{pl}}\cdot \text{PLR}(G)]$$

(5) $$L_{D}=E_{x∼p_{\text{real}}}[\log D(x)]+E_{z∼N(0,1)}[\log(1-D(G(z)))]+\lambda_{R1}∥\nabla D(x){∥}^{2}$$

Among them, *λ*_pl_ = 2 is the weight of the path length regularization term, *λ_R_*_1_ controls the strength of R1 regularization, and PLR refers to the path length regularization term (Path Length Regularization), which is used to maintain the consistency of the generated image style and stabilize the training process.

After the training, we selected the images generated by the last 10,000 steps of the model for FID (Frechet Inception Distance) and Inception Score (IS) as evaluation indicators. The FID metric follows the definition in Equation (6).


(6) $$\text{FID}(x,g)=∥\mu_{x}-\mu_{g}{∥}_{2}^{2}+\text{Tr }({\text{Σ}}_{x}+{\text{Σ}}_{g}-2{\left({\text{Σ}}_{x}{\text{Σ}}_{g}\right)}^{\frac{1}{2}})$$

Where x represents the feature distribution of the real image, and g represents the feature distribution of the generated image. $\mu_{x}$ and $\mu_{g}$ represent the mean vectors of the two distributions, respectively, and Σ*_x_* and Σ*_g_* represent the covariance matrices of the two distributions, respectively. **Tr** represents the trace of the matrix.

Inception Score is a commonly used indicator for evaluating the quality of generated images. It is based on the Inception-v3 model and evaluates the diversity and authenticity of images by calculating the classification probability distribution of generated images. The IS metric follows the definition in Equation (7).


(7) $$\text{IS}=\text{exp }(E_{x}[D_{\text{KL}}(p(y|x)∥p(y))])$$

Where $D_{KL}$ is the KL divergence, p(y|x) is the conditional probability distribution of the classifier output given an image x, and p(y) is the average category distribution of the image.

**Figure 7:**
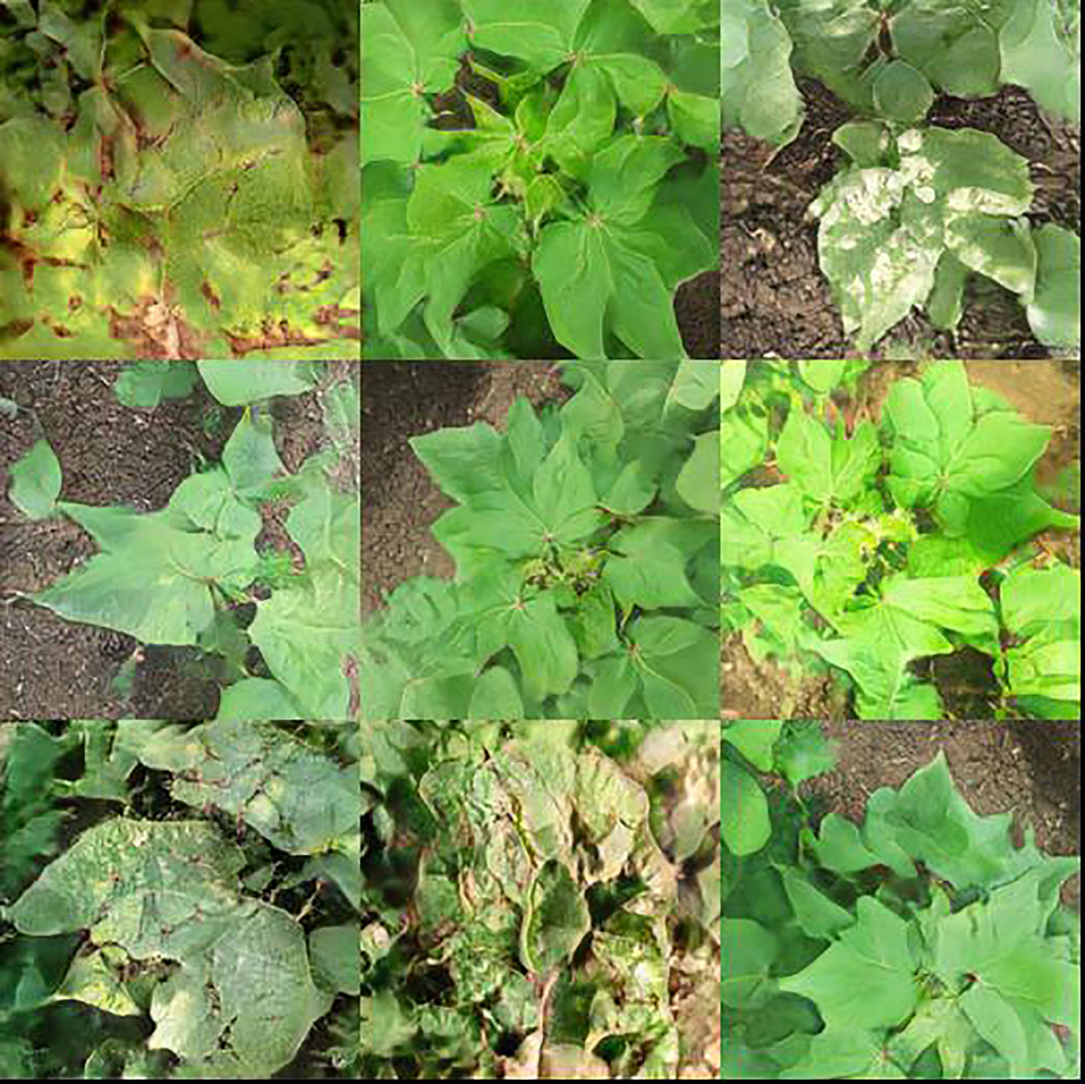
Cotton leaf disease examples generated by StyleGAN-XL.

StyleGAN-XL outperforms other mainstream generative models in both FID and IS indicators as shown in **Table 5**, indicating that it has significant advantages in distribution consistency, structural fidelity, and perceptual quality. The visualization results of the generated samples are shown in **Figure 7**, which further demonstrates its excellent ability to restore the blurred edges of leaf spots and the details of gray mold hairs.

**Table 3:** FID and IS scores of StyleGAN-XL.

GAN model	FID ↓	IS ↑
PGGAN	19.135	6.54
VQ-VAE-2	12.308	7.17
BigGAN	16.318	7.84
StyleGAN-XL	**9.187**	**7.98**

Bold values denote the best performance in each column (lower is better for FID; higher is better for IS). Ties, if any, are all shown in bold.

**Table 4:** Per-category image and instance counts in the final dataset (multi-label).

Category	Images w/class	Instances	Train/val/test (images)
Aphids	1476	1842	1033/296/147
Armyworm	1421	1717	995/284/142
Leaf spot	1488	1963	1041/297/150
Helminthosporium leaf spot	1432	1856	1005/288/139
Fusarium wilt	1408	1774	988/281/139
Boll gray mold	1446	1917	1016/291/139
Leaf curl disease	1329	1658	935/265/129

**Table 5:** Definition of target detection categories for cotton diseases and pests.

Category name	Category type	Feature description
Aphids	Pests	Extremely small targets with strong aggregation
Armyworm	Pests	Dark gray color, nocturnal habits
Leaf spot	Diseases	Multiple circular patches with blurred edges
Bacterial blight	Diseases	Yellowing leaf margins with gradual spread
Fusarium wilt	Diseases	Entire leaf shriveled with collapsed veins
Grey mold	Diseases	Gray-white fuzzy patches with central rot
Leaf curl	Diseases	Twisted and curled leaves, significantly influenced by temperature

The generated samples were evaluated by experts, confirming that they have similar performance to real images in terms of texture structure, color distribution and disease identifiability. Finally, about 2,000 high-quality pseudo images were selected to be added to the original training set, and some of the blurred samples were removed.

The final dataset consists of 10,000 images, comprising 2,000 high-quality pseudo images generated and screened by experts, and 8,000 collected real images. The dataset is divided into training, validation, and test sets in a ratio of 7:2:1, ensuring both sufficient data for model training and reliable performance evaluation (**Table 3**). For annotation, seven types of cotton pests and diseases are used as multi-category detection labels: Aphids, Armyworm, Leaf spot, Helminthosporium leaf spot, Fusarium wilt, Bollgray mold, and Leaf curl disease.

To make the dataset composition clearer under multi-label conditions, we additionally report, for each category, both the number of images that contain at least one instance of the category (“images w/class”) and the total number of annotated instances (“instances”) (**Table 4**). Note that the sum of “images w/class” across categories exceeds 10,000 because multiple categories may appear in the same image.

### 5.2 YOLOv11 model structure and optimization design

In intelligent agricultural systems, object detection serves not only as the first line of pest and disease recognition but also as the perceptual gateway for downstream semantic reasoning and decision support. Unlike traditional image classification, object detection models must both localize and identify multiple objects within a single image. In cotton field applications, this task is further complicated by background clutter, high target density, and the coexistence of objects at various scales Mo and Wei (2024), imposing strict requirements on model robustness, detection accuracy, and real-time responsiveness.

**Figure 8:**
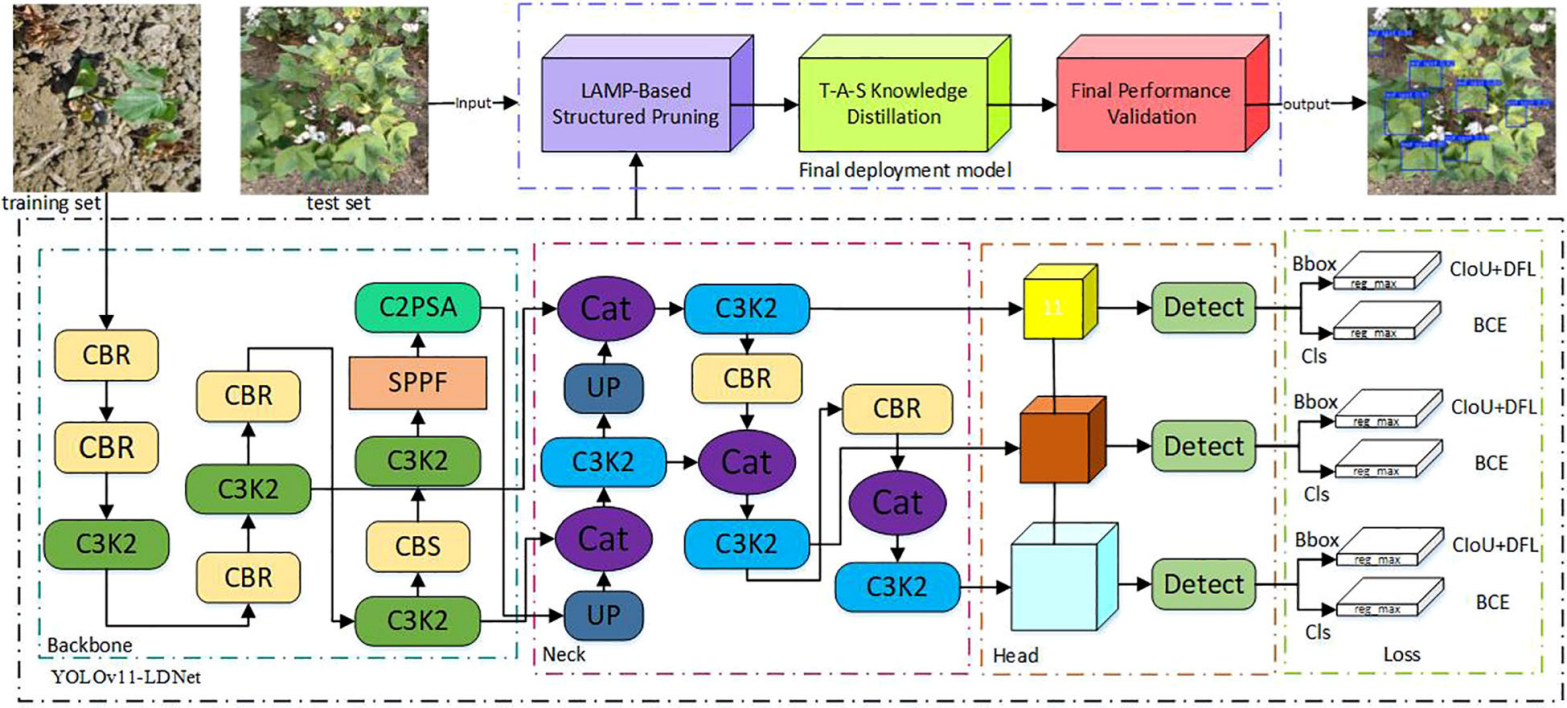
Architecture of the YOLOv11-LDNet.

To address these challenges, this study developed a cotton pest and disease detection model, YOLOv11 LDNet (YOLOv11-LAMP-pruned & Distilled Network), based on the YOLOv11 architecture. The network structure is illustrated in **Figure 8**. On this basis, a series of architectural optimizations and deployment adaptations were introduced to meet the requirements of agricultural field scenarios, resulting in a solution specifically tailored for small-object detection on edge devices and capable of handling multiple pest and disease categories.

YOLOv11, the latest generation of the YOLO series developed by Ultralytics, introduces a series of architectural refinements aimed at achieving a better trade-off between accuracy and efficiency across a wide range of visual tasks, including object detection, instance segmentation, and pose estimation. The backbone incorporates the newly designed C3k2 modules to enhance feature reuse while reducing computational overhead, combined with the SPPF (Spatial Pyramid Pooling – Fast) layer to improve receptive field coverage without significantly increasing inference latency. In addition, the architecture integrates the C2PSA (Convolutional block with Parallel Spatial Attention) mechanism, which strengthens global–local feature interactions and improves robustness in cluttered scenes. The detection head remains decoupled for classification, objectness scoring, and bounding box regression, thereby improving convergence stability and performance in multi-object environments. Compared to YOLOv8, YOLOv11 demonstrates higher mAP with fewer parameters, making it suitable for deployment on both high-performance servers and resource-constrained edge devices.

### 5.3 Category settings and data labeling system

In this study, a specialized seven-class object detection system was developed to address real-world scenarios of cotton field pest and disease identification, covering two major groups: insect pests (aphids and armyworms) and diseases (leaf spot, leaf blight, Fusarium wilt, gray mold, and leaf curl). Each target class presents distinct visual characteristics: aphids, for example, are extremely small, tend to cluster, and exhibit colors similar to the leaf background, making them challenging to detect; leaf blight appears as diffuse, blurry-edged lesions that can cover substantial areas of the leaf surface; leaf curl is marked by significant geometric deformation, requiring shape-aware modeling. To boost recognition accuracy for such diverse targets, all training images underwent meticulous, frame-level annotation following the YOLO standard. A consistent and unified labeling system, detailed in **Table 5**, was established to support precise detection in multi-target and fine-grained settings.

In this research, based on actual cotton field pest and disease scenarios, we have constructed a highly targeted seven-category object detection system covering two major classes: insect pests and diseases. Insect pests include aphids and armyworms, while diseases encompass leaf spot disease, leaf blight, Fusarium wilt, gray mold, and leaf curl disease. These targets exhibit significant differences in their image presentations: for instance, aphids are extremely small and often gather in groups.

### 5.4 LAMP model pruning

In practical deployments of deep neural networks, especially on edge platforms like Jetson Xavier NX, redundant model structures can easily become bottlenecks, restricting both real-time inference and energy efficiency. Although the standard YOLOv11 model delivers high detection accuracy, its substantial parameter count and computational burden limit its usability in resource-constrained agricultural scenarios. To overcome these limitations, this study employs a global pruning technique—Layer-Adaptive Magnitude-based Pruning (LAMP) Notomi et al. (2015). LAMP evaluates the significance of weights across the network and adaptively removes less important parameters layer by layer, while retaining crucial structural features. This global, layer-adaptive pruning approach is driven by the magnitude of weights: each weight’s importance is quantified by its LAMP score (see Equation 8), and pruning is based on normalized importance scores. Through this mechanism, LAMP prevents the “layer collapse” issue observed in conventional pruning and maintains a dynamic trade-off between model compactness and detection accuracy.


(8) $$\text{score}(u;W):=\frac{{\left(W[u]\right)}^{2}}{\sum_{v\geq u}{\left(W[v]\right)}^{2}}$$

**Figure 9:**
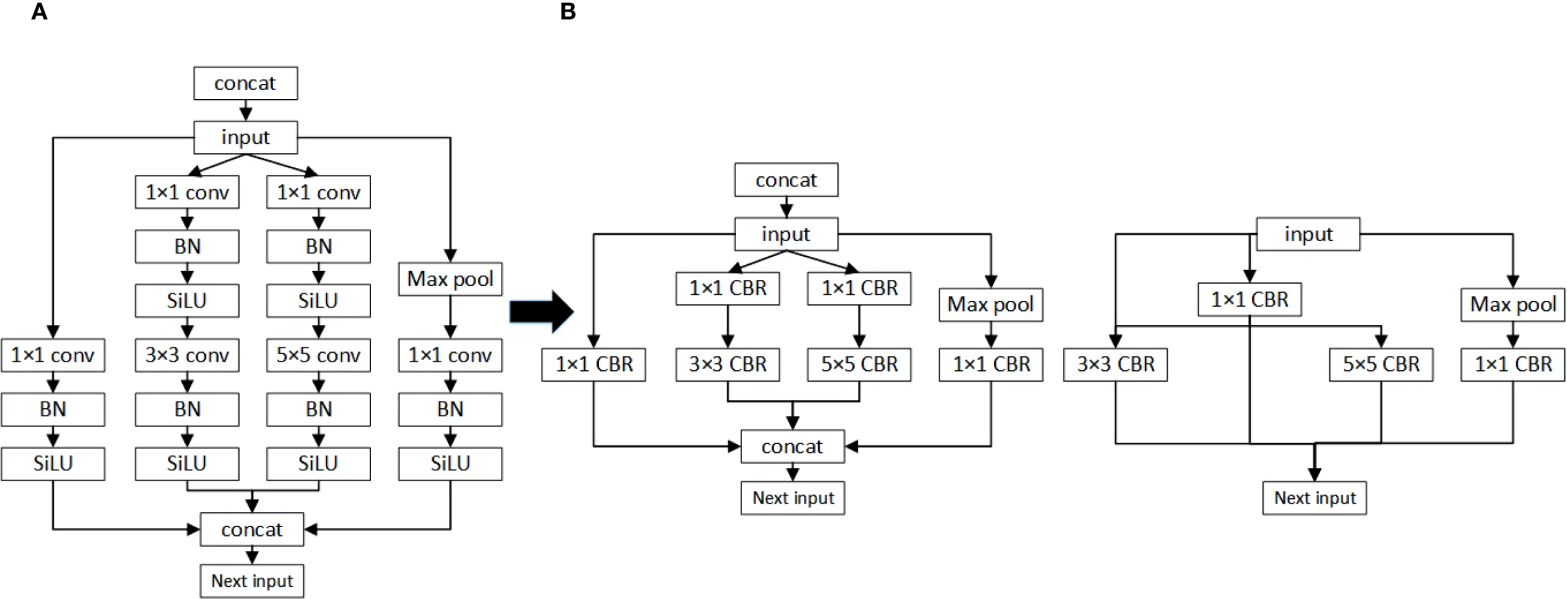
Schematic diagram of the LAMP score calculation process and its application to global pruning. **(A)** Vertical fusion **(B)** Horizontal fusion.

In the LAMP approach, the numerator (*W*[*u*])^2^ represents the squared value of an individual weight, while the denominator sums the squares of all weights from the current position to the end of the layer. This method accounts for both the absolute size of each weight and its relative significance within the layer, providing an effective measure of importance. The pruning rule is straightforward: if (*W*[*u*])^2^ *>* (*W*[*v*])^2^, then *W*[*u*] has a higher LAMP score and is deemed more important than *W*[*v*]. LAMP applies a global sparsity threshold and iteratively removes weights with the lowest scores until the target is achieved, ensuring that at least one key connection remains in every layer to prevent collapse. The overall strategy is depicted in **Figure 9**. This adaptive, global pruning scheme significantly reduces model complexity and resource usage, supporting lightweight deployment in challenging operational environments.

### 5.5 Knowledge distillation

In real-world cotton pest and disease detection, the presence of cluttered backgrounds, indistinct lesion boundaries, and small insect bodies in images makes accurate discrimination particularly challenging for models. These issues are even more pronounced when models are deployed on resource-constrained devices like Jetson Xavier NX, where maintaining a trade-off between accuracy and model size is essential. While standard compression methods such as pruning can lower parameter counts and computation needs, they often result in nonlinear information loss, leading to a noticeable drop in detection performance for fine-grained features Lin et al. (2022).

**Figure 10:**
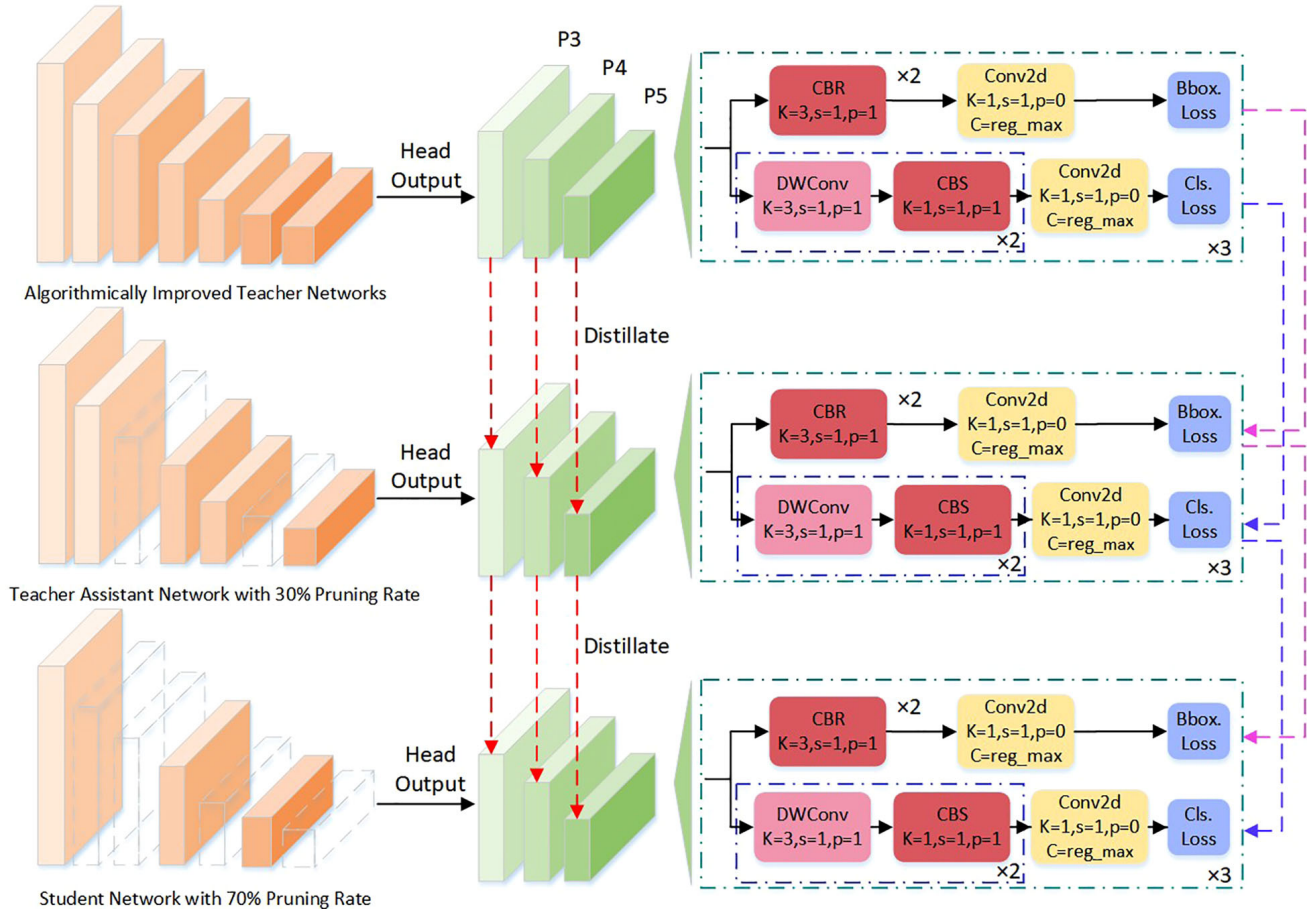
Knowledge distillation network structure diagram.

To address these challenges, this paper adopts a hierarchical collaborative knowledge transfer approach—the Teacher-Assistant-Student Knowledge Distillation (TAS-KD) framework—as a core strategy to recover model performance following pruning. The key idea is to gradually transfer the rich representation abilities of a large teacher network to a compact student model, using an intermediate assistant network to mediate knowledge flow and maintain architectural consistency. This setup allows for more effective and flexible distillation, as illustrated in **Figure 10**, which details the specific stages of the process.

Unlike standard distillation techniques, this work constructs a ternary cognitive transfer scheme: the teacher model provides comprehensive multi-scale semantic representations as the source of information; the assistant network bridges differences in capacity and task representation, functioning as both a mediator and a semantic compressor; the student model focuses on lightweight structure while learning from the others, with deployability as a key target. By using this multi-stage distillation, the approach narrows the gap in representational ability between models, helping the student achieve better generalization and fine-grained perception even with strict parameter limits.

In the actual implementation, this study adopts a Soft Target Transfer approach as the main means for knowledge delivery. Beyond producing class predictions, the teacher model outputs high-dimensional class distribution vectors that encode implicit relationships—such as similarities among categories, background context, and the relative positions of targets. These soft labels carry more information than standard one-hot (hard) labels and help guide the student model in addressing challenging distinctions in cotton pest and disease images, including indistinct leaf boundaries, overlapping lesions, and occluded insect bodies.

To further strengthen the preservation of spatial structure, the assistant model not only passes on category-level semantics but also transfers spatial attention maps for each target. By distilling knowledge through both category and location channels, this approach enables the student model to learn contextual relations among targets and maintain boundary consistency during training. As a result, the student gains improved robustness and flexibility, especially in handling common field challenges like crowded scenes, small objects, and ambiguous class boundaries.

The improved YOLOv11 and its pruned version share the same network architecture, differing only in the number of channels. Both heads generate three feature maps, so the knowledge distillation process illustrated in **Figure 8** applies equally to both. During training, the teacher and student models operate in parallel, with losses computed on each feature map. For this purpose, L2 loss is adopted, as detailed in Equation 9.


(9) $${\text{Loss}}_{\text{dis}-\text{feature}}=\frac{\sum_{i=1}^{n}{\left(F_{i}(i)-F_{s}(i)\right)}^{2}}{n}$$

In the formula, *F_t_* (·) represents the feature map of the teacher network, and *F_s_* (·) represents the feature map of the student network.

In addition to the loss on the feature map, the paper also performs distillation learning on the classification and regression losses. Let s be the output of the student network, t be the output of the teacher network, and the regression distillation loss uses L2 loss, see Equation 10. The classification distillation loss uses cross entropy loss, see Equation 11.


(10) $${\text{Loss}}_{\text{dis}-\text{reg}}=\frac{\sum_{i=1}^{n}{\left(Reg_{i}(i)-Reg_{s}(i)\right)}^{2}}{n}$$

(11) $${\text{Loss}}_{\text{dis}-\text{cls}}=-\sum_{i=1}^{n}{\text{ClS}}_{i}(i)\text{log }({\text{ClS}}_{i}(i))$$

Combining the distillation loss and the actual student network loss is the final student network loss. The calculation formula is shown in Equation 12, where the original loss of the student network is Loss.


(12) $$\text{Loss}={\text{Loss}}_{\text{stu}}+\delta \;{\text{Loss}}_{\text{dis}-\text{feature}}+\zeta \;{\text{Loss}}_{\text{dis}-\text{cls}}+\epsilon \;{\text{Loss}}_{\text{dis}-\text{reg}}$$

This method is of great significance for the improvement of lightweight models, making them more suitable for deployment on resource-constrained mobile devices, and providing technical support for the rapid detection and prevention of cotton leaf diseases.

## 6 Model training and results analysis

### 6.1 Experimental environment configuration

To comprehensively assess cotton pest and disease monitoring and diagnostic methods, this research developed a real-time monitoring platform that emphasizes both efficiency and interactivity. The platform integrates deep learning-based object detection with knowledge graph reasoning, enabling accurate identification and real-time feedback for cotton pest and disease cases. Initial model training and optimization were performed on a system equipped with a GeForce RTX 4090 GPU and running Ubuntu 18.04 LTS. The development environment consisted of PyCharm 2020.3, Python 3.7.0, PyTorch 1.7.1 for deep learning, and OpenCV 3.4.6 for image preprocessing.

To meet the deployment demands of resource-limited agricultural environments, this study employed LAMP pruning and a teacher–assistant–student knowledge distillation framework, making the YOLOv11n model both efficient and lightweight, and greatly reducing requirements for computation and storage. The resulting optimized model was successfully implemented on the NVIDIA Jetson Xavier NX platform, striking a favorable balance between power efficiency and performance Kortli et al. (2022). Meanwhile, the system’s knowledge graph reasoning module, built with Neo4j, encompasses information on cotton pest and disease categories, pathogens, transmission factors, symptoms, and prevention strategies, enhancing the accuracy and intelligence of reasoning. The graph contains over 5,000 nodes and relationships, supporting multi-dimensional associations for comprehensive diagnosis in complex conditions.

**Figure 11:**
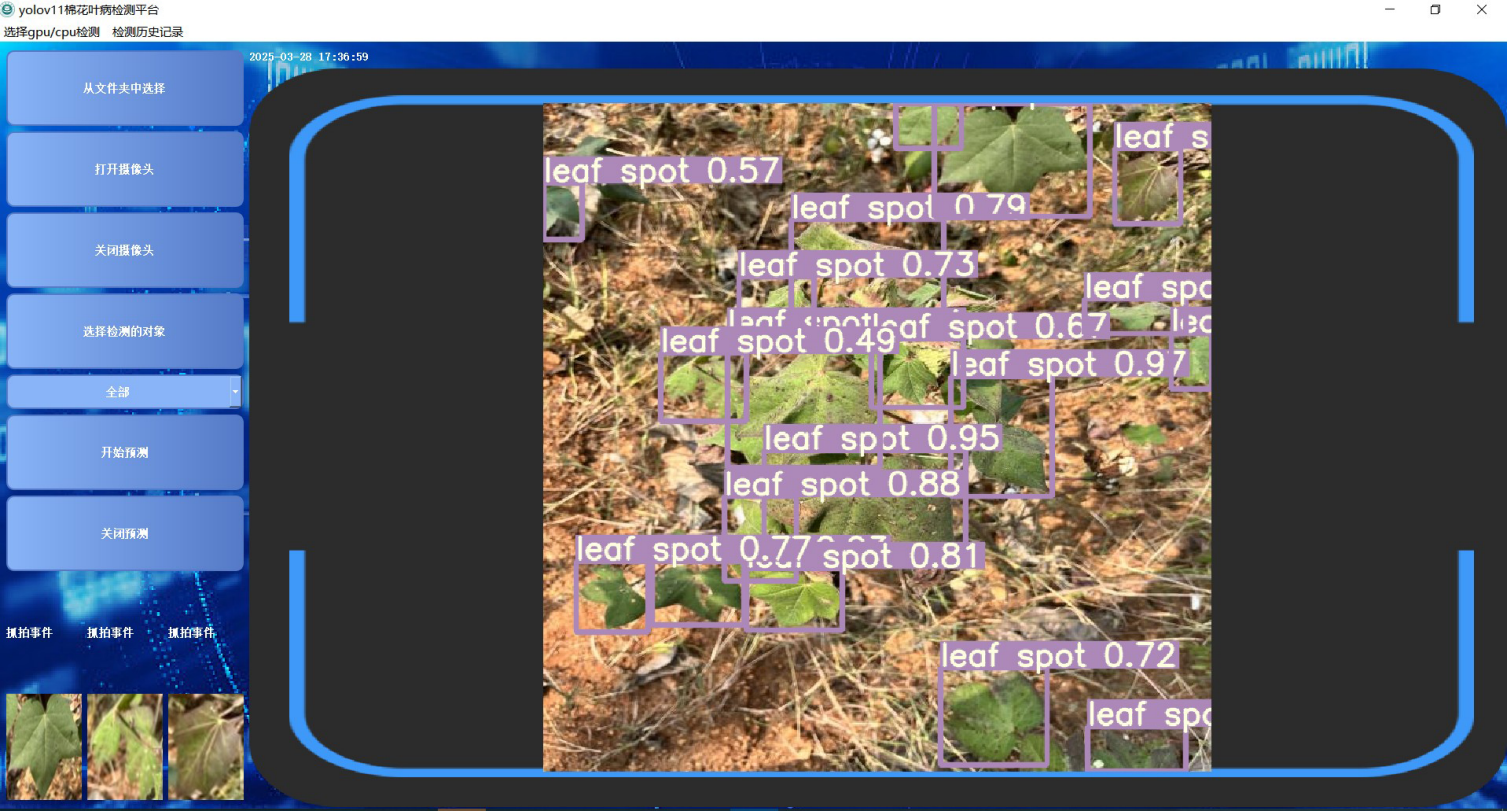
Detection system interface.

To improve user interaction and system usability, the platform features a real-time interactive interface where users can directly view pest and disease detection results along with relevant knowledge graph data. The interface presents details such as disease type, confidence score, transmission routes, and prevention recommendations. Users are also able to give immediate feedback and log their actions as needed. An example of the interface is shown in **Figure 11**.

### 6.2 Model training parameter settings

In this work, stochastic gradient descent (SGD) is adopted as the optimizer, with the momentum parameter set at 0.937 to improve optimization stability and efficiency Gower et al. (2019). The initial training phase consists of 200 epochs with a learning rate of 0.01, allowing the model to rapidly converge toward a viable solution. As training progresses and the model nears its optimal state, the learning rate is reduced to 0.0001 over the subsequent 150 epochs for performance fine-tuning. To guard against overfitting, a weight decay (L2 regularization) of 0.0005 is incorporated. Additionally, to address potential instability caused by weight initialization, a warm-up strategy is used, where the learning rate starts lower for the first three epochs and gradually increases to the preset value, helping mitigate early-stage training difficulties.

### 6.3 Experimental results and analysis

To thoroughly assess the real-world performance of the proposed intelligent cotton pest and disease recognition and voice interaction system, we deployed the trained and optimized YOLOv11 model—after applying LAMP pruning and hierarchical knowledge distillation—on the Jetson Xavier NX edge platform. The system was then tested and demonstrated in an actual agricultural field setting to evaluate its effectiveness and operational performance.

To further evaluate the real-time performance of the system across various image resolutions and concurrent input streams, additional experiments were conducted. On the Jetson Xavier NX platform, increasing the input resolution from 640×640 to 1280×1280 reduced inference speed from 52 FPS to 24 FPS, illustrating a clear balance between image quality and processing speed. When handling two image streams at once, the per-stream frame rate dropped by about half, primarily due to limitations in GPU memory and bandwidth. These findings show that, while the system can achieve real-time detection with single high-resolution inputs, performance is affected in multi-stream or ultra-high-resolution settings—factors that must be considered for deployment. Future efforts will focus on optimizing parallelism and resource management to improve performance in multi-stream scenarios.

To further address the reviewer’s feedback, we benchmarked the system on several representative edge devices: NVIDIA Jetson Nano, Jetson Xavier NX, and a desktop PC (Intel i7-12700 + RTX 4060). **Table 6** shows the inference speeds (FPS) for the optimized YOLOv11n model with 640×640 single-stream input on each platform. The Jetson Xavier NX delivered real-time results at 52 FPS, Jetson Nano achieved 12 FPS, and the desktop PC exceeded 120 FPS. These outcomes demonstrate the method’s scalability, while also indicating that further model compression and acceleration are needed for deployment on more limited devices like Jetson Nano or Raspberry Pi. Future work will expand evaluations to other platforms and explore ultra-lightweight deployment options.

**Table 6:** Inference speed of YOLOv11n on different devices (640×640, single stream).

Device	FPS (frames per second)	Notes
Jetson Xavier NX	52	Real-time
Jetson Nano	12	Acceptable, lower power
Desktop PC (i7+RTX4060)	*>*120	High-end, reference only

**Figure 12:**
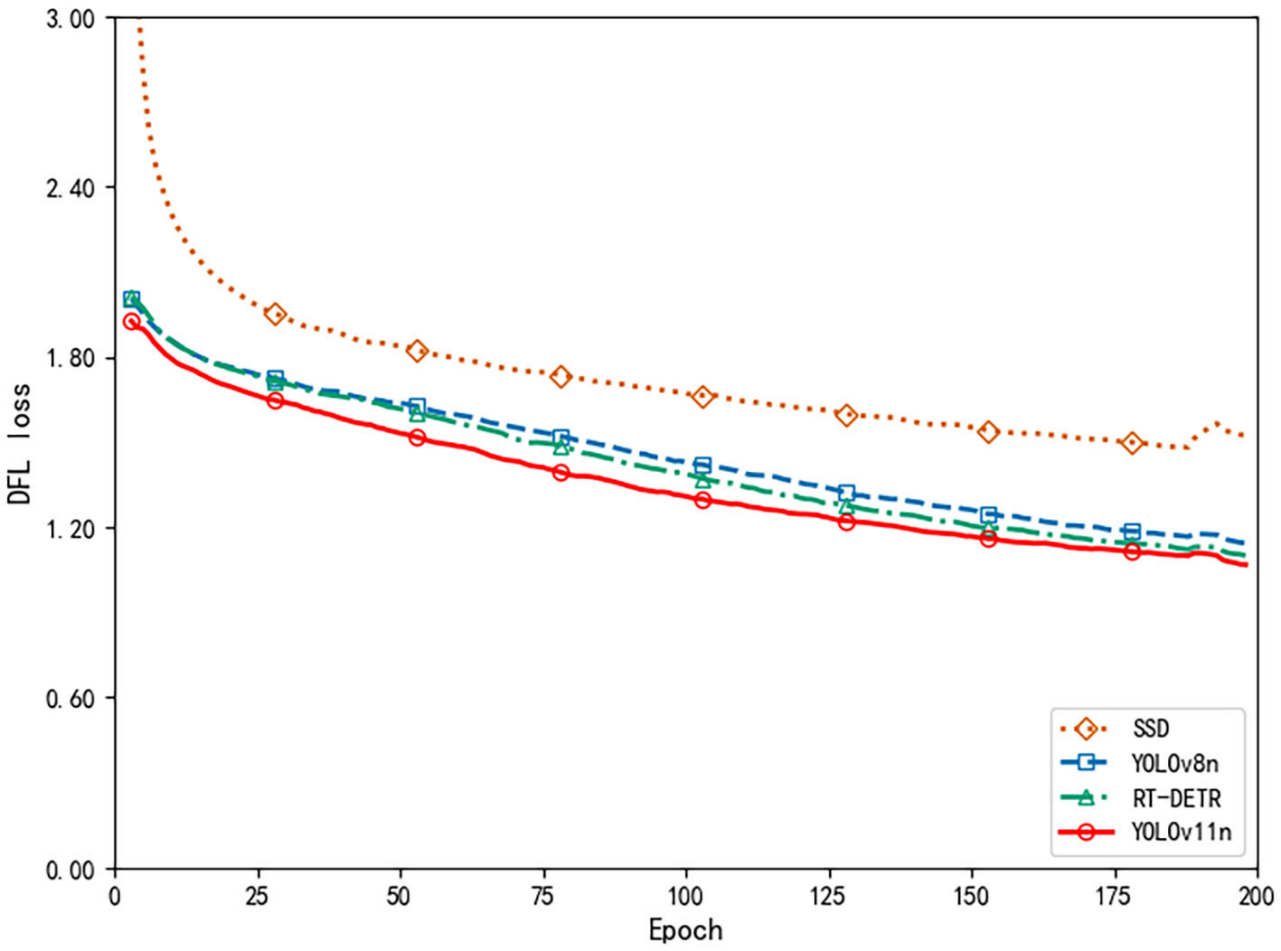
Average precision comparison.

Further examination of the training process reveals, as depicted by the mAP_50_ curve in **Figure 12**, that model accuracy improved swiftly during the initial training phase, reflecting effective acquisition of fundamental pest and disease features. After about 100 epochs, mAP_50_ plateaued near 85%, indicating that the model had successfully captured the essential patterns in complex cotton pest and disease scenarios and had entered a stable, optimal training state in the later stages.

**Figure 13:**
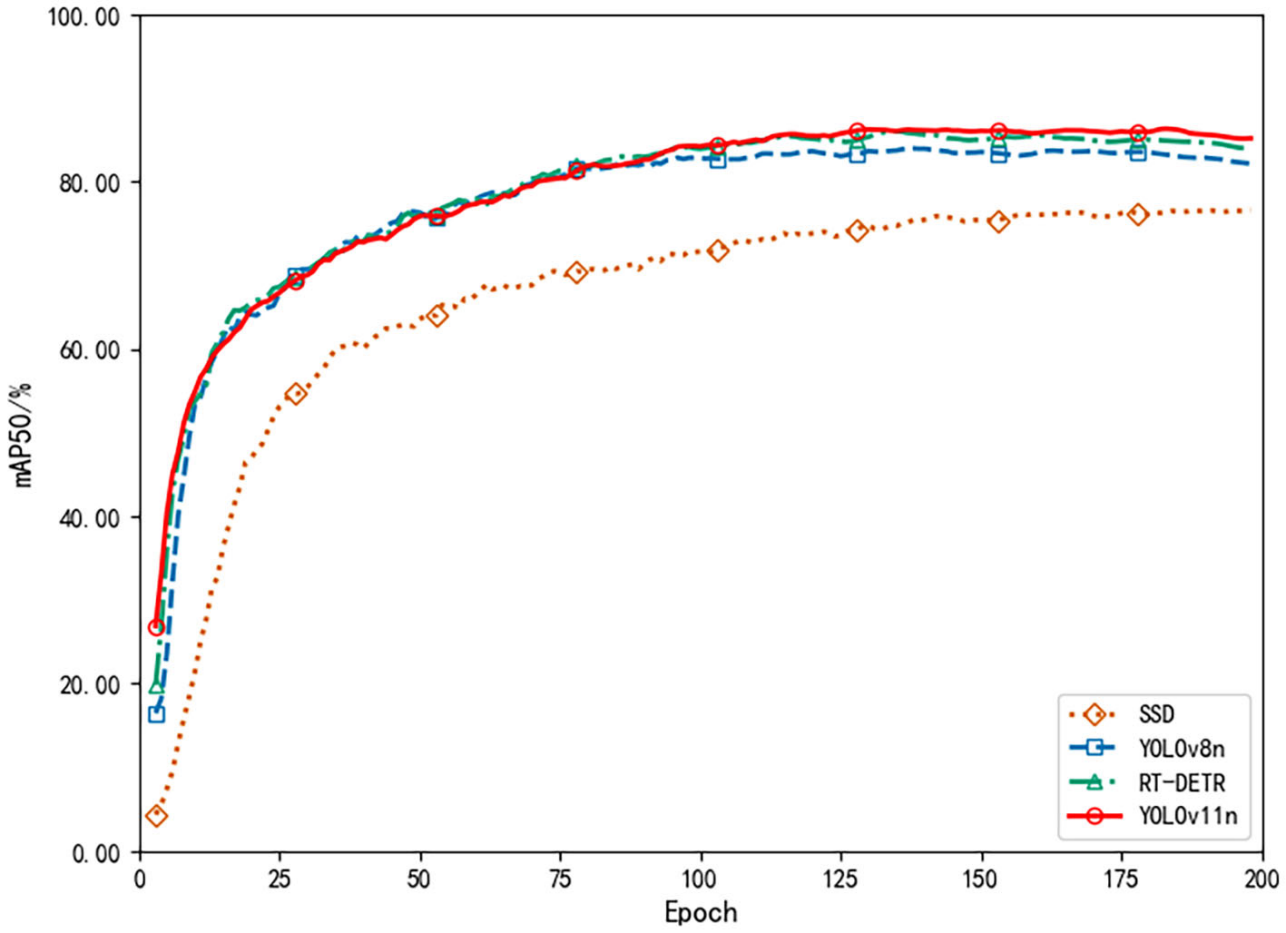
DFL loss comparison.

The DFL loss curve in **Figure 13** provides additional evidence supporting this trend. During the early stages of training, the loss dropped sharply, indicating rapid improvement in the model’s ability to localize and classify pest and disease targets. After 50 epochs, the rate of loss reduction slowed but continued to decline steadily, demonstrating that the model was still optimizing its detailed recognition abilities. Ultimately, the loss reached a relatively low value, confirming the model’s solid convergence and effective optimization throughout training.

To further verify the performance of the proposed YOLOv11n model, we compared multiple model variations and state-of-the-art benchmarks, including SSD Liu et al. (2016b), YOLOv8n Jocher et al. (2023), and RT-DETR Zhang et al. (2023). **Table 7** presents the results: the YOLOv11n model delivers the highest values for average accuracy (87.42%), recall (85.19%), and mAP_50_ (85.14%), while also offering the smallest model size (5.3 MB) and the fastest inference time (0.022 s). These findings indicate that, relative to other leading frameworks, YOLOv11n achieves a strong balance of efficiency and precision, making it especially suitable for real-time use in resource-limited agricultural settings. In **Table 7**, “YOLOv11n (Ours)” refers to the original baseline model (5.3 MB), whereas in **Table 8**, “YOLOv11n (70% pruned+distilled)” is the final optimized model (1.3 MB) used for system deployment.

**Table 7:** Comparison of indicators of different models.

Model	Average accuracy (%)	Average recall (%)	mAP_50_ (%)	Weight file size (MB)	Recognition time (s)
SSD	77.97	74.37	76.86	90.3	0.078
YOLOv8n	85.88	82.35	83.54	6.2	0.026
RT-DETR	86.76	84.11	84.20	64.9	0.085
YOLOv11n (Ours)	**87.42**	**85.19**	**85.14**	**5.3**	**0.022**

Bold values denote the best performance in each column (higher is better for Average accuracy, Average recall, and mAP; lower is better for weight file size and recognition time). Ties, if any, are all shown in bold.

**Table 8:** Performance comparison of different YOLOv11n models on wheat pest and disease detection.

Model	Avg. accuracy (%)	Avg. recall (%)	${\text{mAP}}_{50}$ (%)	Params (M)	GFLOPs	Weight size (MB)	Inference time (s)
YOLOv11n (no pruning)	87.42	85.19	85.14	2.5	6.3	5.3	0.022
YOLOv11n (30% pruned)	86.37	81.42	84.76	1.0	3.6	4.0	0.021
YOLOv11n (70% pruned)	82.45	77.68	81.13	0.4	1.8	1.3	0.019
YOLOv11n (70% pruned+distilled)	87.05	**84.20**	**86.10**	**0.4**	**1.8**	**1.3**	**0.019**

Bold values denote the best performance in each column (higher is better for Average accuracy, Average recall, and mAP50; lower is better for Params, GFLOPs, weight size, and inference time). Ties, if any, are all shown in bold.

In conclusion, the cotton pest and disease intelligent recognition and voice interaction system developed in this study demonstrated strong recognition accuracy and reliability in real-world settings. By employing model pruning and knowledge distillation, the system achieved an effective balance between real-time performance and resource utilization. These strengths provide robust intelligent decision support for agricultural production and highlight the system’s significant potential for broader application and dissemination.

### 6.4 Pruning ablation study

To thoroughly assess the effects of various pruning strategies on model performance, we conducted a comparative analysis of LAMP pruning and channel pruning at compression rates of 30% and 70%. As detailed in **Table 8**, all models were evaluated on the tea pest and disease detection dataset, utilizing the same YOLOv11n backbone and identical experimental conditions.

The findings indicate that LAMP pruning consistently outperforms channel pruning in terms of average accuracy, recall, and mAP at both 30% and 70% pruning levels. Notably, as the pruning rate increases to 70%, the advantage of LAMP becomes even more significant. This approach better preserves essential network connections and key features, leading to greater detection accuracy and stability, especially when models are highly compressed. These results suggest that LAMP pruning is particularly well-suited for lightweight detection model deployment in resource-constrained agricultural settings.

Overall, these results indicate that LAMP pruning provides superior performance under both moderate and aggressive compression rates, making it a robust and practical solution for lightweight deployment of tea pest and disease detection models in edge computing scenarios.

### 6.5 Knowledge distillation ablation study

On top of model pruning, we further investigated the effect of different knowledge distillation strategies on the performance of compact models. Specifically, we compared the conventional direct knowledge distillation approach with the Teacher-Assistant-Student (TAS) three-stage distillation method, both applied to the 70% pruned YOLOv11n model. The results are summarized in **Table 9**.

**Table 9:** Performance comparison of different knowledge distillation strategies applied to the 70% pruned YOLOv11n model.

Model	Avg. accuracy (%)	Avg. recall (%)	mAP_50_ (%)	Params (M)	GFLOPs	Weight size (MB)	Inference time (s)
70% Pruned YOLOv11n + Direct Distillation	85.82	82.11	84.73	0.3	1.5	1.1	0.019
70% Pruned YOLOv11n + TAS Distillation	88.96	83.58	85.31	0.3	1.5	1.1	0.019

The TAS distillation framework incorporates an assistant model between the teacher and student, allowing knowledge to be transferred incrementally through multiple stages. Experimental results demonstrate that this approach markedly improves average accuracy, recall, and mAP compared to direct distillation, and effectively compensates for performance drops due to heavy pruning. These findings confirm that the three-stage distillation process boosts the generalization and detection abilities of pruned lightweight models, offering a robust solution for agricultural scenarios where both compactness and accuracy are required.

### 6.6 Model detection effects

**Figure 14:**
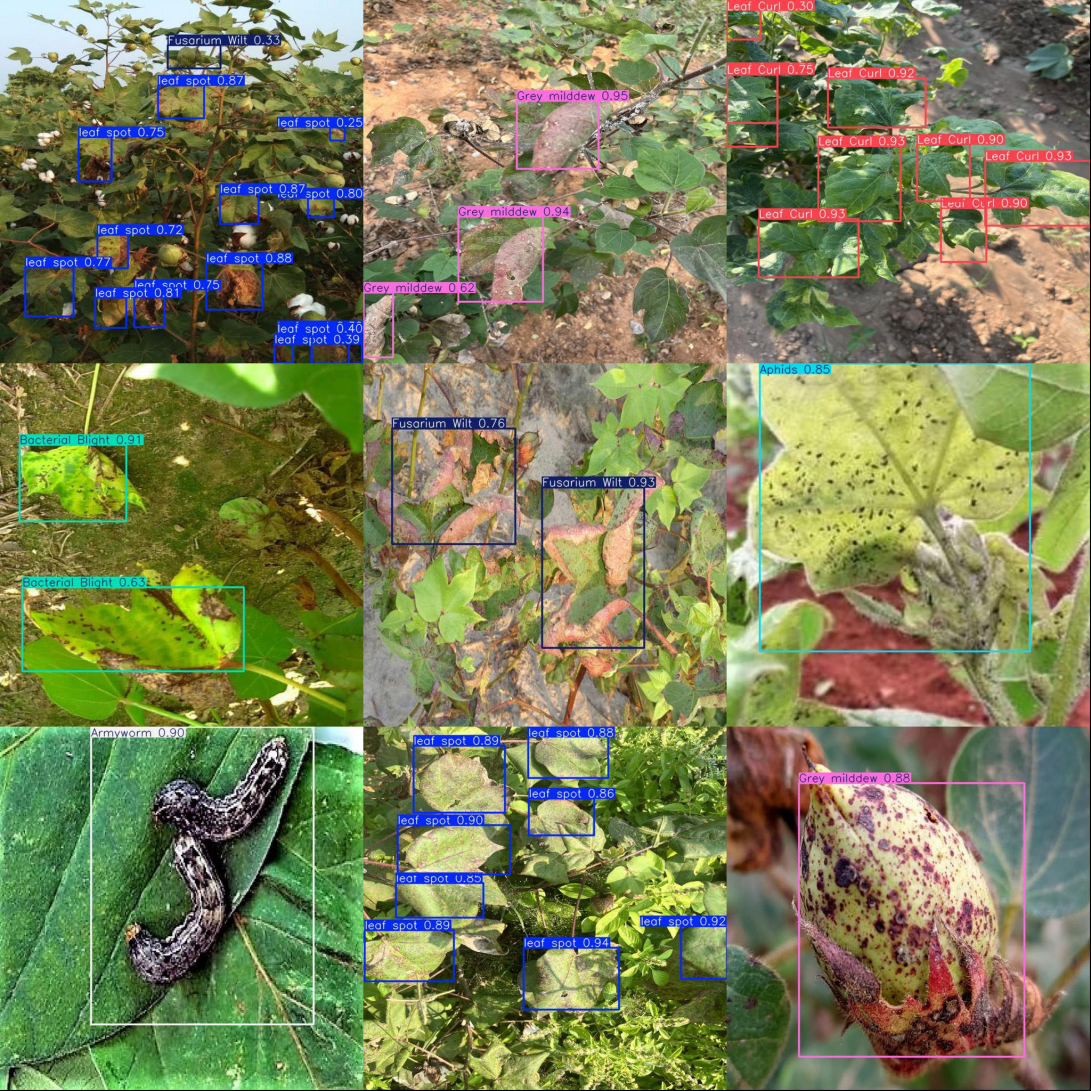
Detection results for different object categories.

To assess the practical effectiveness of the cotton pest and disease intelligent recognition and voice interaction system, we deployed the improved YOLOv11-LDNet model on the Jetson Xavier NX edge platform and carried out real-time detection in actual cotton fields. **Figure 14** illustrates the system’s detection results across various categories of cotton pests and diseases in real-world field settings.

During field testing, the system reliably identified and classified different cotton pests and diseases. For leaf spot, the model precisely pinpointed lesion areas and assigned high confidence scores, indicating strong feature sensitivity and localization accuracy. For small insect pests like aphids and armyworms, the system maintained accurate detection and high confidence even in cluttered backgrounds. The platform also performed well with gray mold, Fusarium wilt, leaf blight, and leaf curl, highlighting that the improved YOLOv11-LDNet, combined with LAMP pruning and knowledge distillation, sustains robust detection performance while enabling efficient deployment.

Detection outcomes are delivered to farmers in real time via Bluetooth speakers, with the system leveraging the knowledge graph to provide detailed explanations of identified diseases and targeted prevention advice. This workflow demonstrates the practical benefits and efficiency of the solution in real agricultural production, further validating the system’s effectiveness and feasibility for intelligent pest and disease recognition and management.

Both demonstration results and measured performance data confirm that the proposed cotton pest and disease intelligent recognition and voice interaction system offers strong prospects for field application. The system helps lower the barrier of professional expertise and reduces manual labor costs, significantly enhancing the speed and efficiency of disease prevention and control in agricultural operations.

## 7 Cotton pest and disease intelligent Q&A system

**Figure 15:**
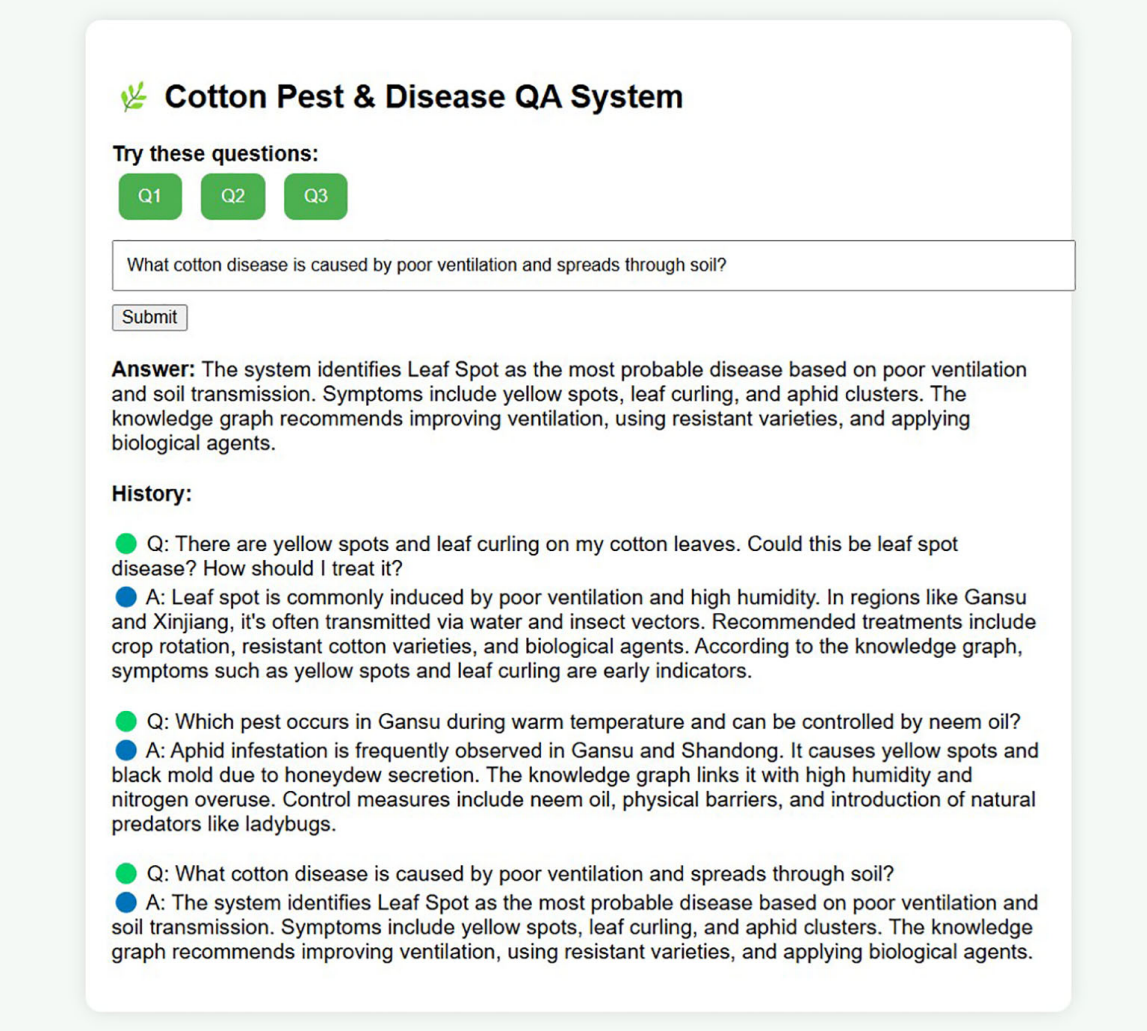
Cotton pests and diseases knowledge graph driven question-answering interface.

To improve interactivity and user experience, this research developed a knowledge graph-based intelligent Q&A system for cotton pests and diseases. Leveraging the Neo4j-stored knowledge graph as the core data source, the system utilizes natural language processing to deliver real-time semantic understanding and accurate answers to pest and disease queries Yu et al. (2024). In experimental testing, a range of representative farmer questions—such as “How should brown spots on cotton leaves be managed?” and “What pesticides are recommended for leaf blight?”—were randomly selected. The Q&A system rapidly provided diagnostic details and prevention advice, with immediate feedback delivered through voice devices. The experiments showed an accurate response rate exceeding 95%, and average response times under 2 seconds. These results indicate that the Q&A system not only raises the professionalism and convenience of pest and disease diagnosis, but also reduces the operational barrier for farmers, demonstrating considerable potential for widespread agricultural application. **Figure 15** presents the experimental results.

### 7.1 Experimental evaluation of the voice interaction module

To rigorously evaluate the voice interaction feature, a series of controlled experiments were conducted in both laboratory and real field environments.

**Test Setup:** The system was deployed on the Jetson Xavier NX with a Bluetooth-connected portable speaker. Test queries were derived from a curated set of 50 representative farmer questions covering pest identification, symptom description, and management advice. Both single-turn and multi-turn interactions were tested.

**Evaluation Metrics:** The module was assessed along four dimensions: (1) *Speech synthesis clarity* (mean opinion score rated by 15 participants on a 1–5 scale), (2) *Information accuracy* (percentage of voice output matching the correct textual answer from the Q&A system), (3) *Response latency* (time from query submission to voice output start), and (4) *Field intelligibility* (recognition rate by farmers in outdoor conditions with background noise).

**Results:** In indoor tests, the mean opinion score reached 4.6/5, with 98% information accuracy and an average latency of 1.92 s. In outdoor field trials under wind speeds up to 5 m/s and ambient noise levels of 60–70 dB, intelligibility remained above 93%. The Bluetooth range exceeded 10 m without noticeable signal loss.

**User Feedback:** Farmers reported that the voice output reduced the need to check device screens during field operations, improving convenience and operational efficiency.

In conclusion, the cotton pest and disease intelligent identification and voice interaction system developed in this work not only achieved high recognition accuracy and reliability in real-world settings, but also maintained a favorable balance between real-time performance and resource efficiency through the use of model pruning and knowledge distillation. This provides robust intelligent decision support for agricultural production and demonstrates significant potential for widespread adoption and application.

### 7.2 Migration and performance verification of the wheat pest and disease detection system

To comprehensively evaluate the generalizability of the intelligent detection and Q&A system across different crops, we extended our validated approach from cotton pest and disease detection to wheat, addressing 15 common wheat pest and disease categories. A dedicated knowledge graph for wheat was constructed, integrating authoritative publications, expert insights, and major agricultural databases. This structured representation greatly enhanced the accuracy and depth of semantic reasoning for wheat pest and disease scenarios.

We continued to adopt YOLOv11n as the backbone detection model, employing LAMP pruning and knowledge distillation strategies for lightweight deployment. To rigorously evaluate the model’s generalization capabilities and performance, we compared multiple model variations and state-of-the-art benchmarks under realistic conditions. The models were deployed and accelerated via TensorRT on Jetson Xavier NX edge devices.

**Table 10** presents detailed performance comparisons. Our optimized YOLOv11n model (70% pruned and distilled) maintains high accuracy and recall rates while significantly reducing computational complexity and inference latency, demonstrating superior performance compared to baseline and other lightweight models.

**Table 10:** Performance comparison of different YOLOv11n models on wheat pest and disease detection.

Model	Avg. accuracy (%)	Avg. recall (%)	mAP_50_ (%)	Params (M)	GFLOPs	Weight size (MB)	Inference time (s)
YOLOv11n	88.16	83.05	86.29	2.5	6.3	5.3	0.023
YOLOv11n (30% pruned)	86.37	81.42	84.76	1.0	3.6	4.0	0.021
YOLOv11n (70% pruned)	82.45	77.68	81.13	0.4	1.8	1.3	0.019
YOLOv11n (70% pruned+distilled)	87.05	84.20	86.10	0.4	1.8	1.3	0.019

Additionally, to benchmark our model against other state-of-the-art detection frameworks under identical conditions, **Table 11** compares our optimized YOLOv11n model with widely recognized models including SSD, YOLOv8n, and RT-DETR. The results show that our proposed model achieves superior balance in accuracy, speed, and model compactness, proving its suitability for practical agricultural deployment.

**Table 11:** Benchmarking YOLOv11n against other state-of-the-art models for wheat pest and disease detection (15 categories).

Model	Average accuracy (%)	Average recall (%)	mAP_50_ (%)	Weight file size (MB)	Recognition time (s)
SSD	78.54	75.21	77.42	90.3	0.080
YOLOv8n	86.72	83.89	85.20	6.2	0.027
RT-DETR	87.43	84.10	85.95	64.9	0.087
YOLOv11n (Ours, 70% pruned+distilled)	87.05	84.20	86.10	1.3	0.019

**Figure 16:**
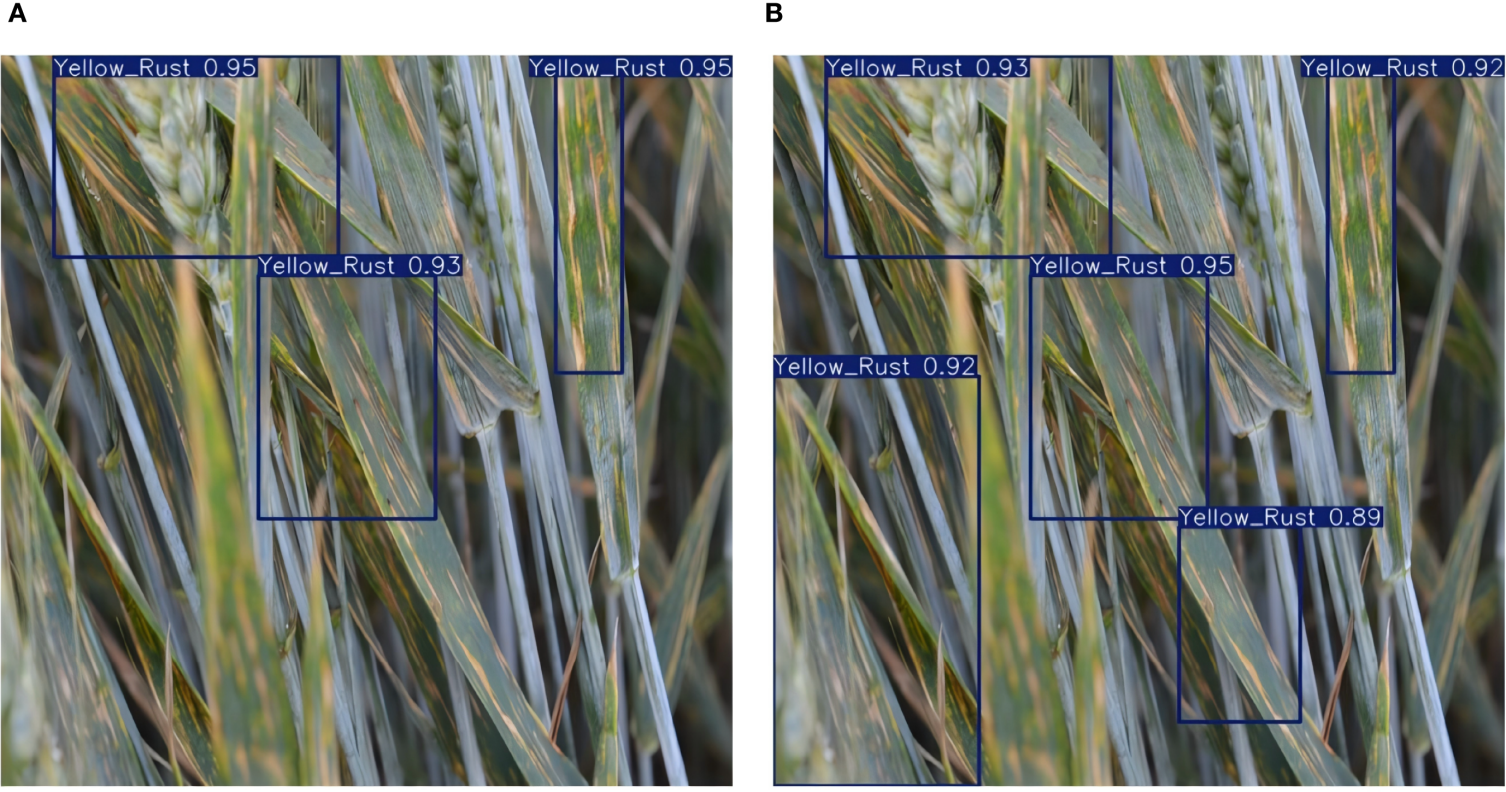
Visualization results of wheat pest and disease detection in complex field scenarios (placeholder).

Furthermore, **Figure 16** shows example visualizations of wheat pest and disease detection in complex field conditions, including situations with changing lighting, heavy occlusion, and cluttered backgrounds. The system consistently delivers high-confidence, accurate multi-class detection results under these challenging circumstances, highlighting its robustness and practical suitability for deployment in a variety of real-world environments.

**Figure 17:**
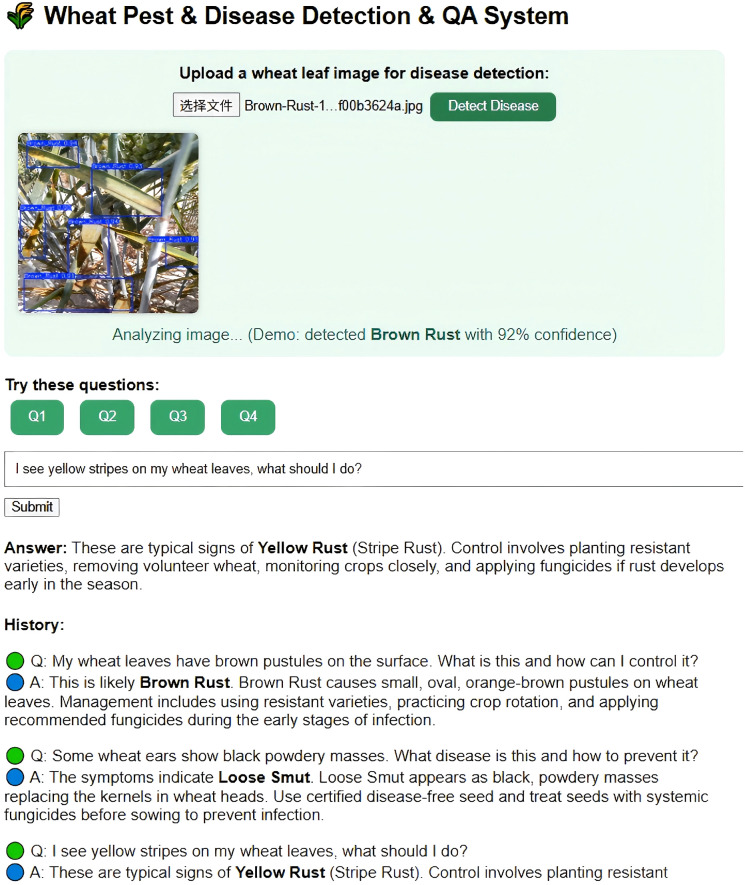
User interface of the wheat pest and disease knowledge graph-based question-answering system (placeholder).

To further improve the system’s interactivity and user experience, a knowledge graph-based intelligent Q&A module for wheat pest and disease management was developed. **Figure 17** shows a representative system interface where users can inquire about symptoms, management approaches, and preventive measures, receiving timely and accurate feedback through both text and voice channels. The Q&A system is designed to support multi-turn conversations and handle complex query types, making it more accessible for farmers and agricultural technicians.

These results and visualization analyses jointly confirm the robust generalization, high accuracy, and practical value of the proposed system for multi-crop, real-world agricultural scenarios.

## 8 Discussion

This research developed an intelligent diagnosis system for cotton pests and diseases, integrating deep learning-based object detection, knowledge graph reasoning, and voice interaction. By refining the YOLOv11-LDNet model and applying LAMP pruning along with knowledge distillation, the system achieves substantial parameter reduction while preserving high detection accuracy and speed, meeting the requirements of limited-resource settings. The knowledge graph captures agricultural knowledge in a structured way and leverages Neo4j for fast reasoning and querying, supporting timely and precise responses to complex and ambiguous issues. The system is suitable for edge deployment and provides 823 immediate user feedback via voice output, greatly enhancing both efficiency and user experience.

A central usability observation from our study is that *multi-label or ambiguous diagnoses* (e.g., simultaneous detection of leaf spot and Fusarium wilt) can confuse users if delivered as a single message. To address this, we now prioritize this finding in our design implications and added sequential message delivery with clear summaries, structured pauses, and explicit uncertainty cues for low-confidence results to improve user understanding. The system also tackled real-world issues like reduced intelligibility from field noise by introducing adaptive volume adjustment and software-based noise reduction. In addition, we exposed user controls for broadcast speed and implemented a roadmap for dialect support to reflect user preferences surfaced in the study. These updates, based on user and expert input, improved the clarity and reliability of the voice interaction; nevertheless, broader field studies are needed to fully confirm its utility across diverse agricultural contexts.

To improve usability and accessibility, particularly for users with limited literacy, a preliminary user study was conducted. Four participants—three front-line cotton farmers with different literacy backgrounds and one plant protection specialist—took part in a structured evaluation under typical field conditions. Each was instructed to use the system independently, relying solely on the voice guidance, and then completed a short questionnaire and semi-structured interview. The survey used a 5-point Likert scale to assess usability, clarity of the voice output, and operational simplicity. Results showed an average usability score of 4.3/5 overall and 4.1/5 for low-literacy users. We explicitly acknowledge that this pilot sample (*n* = 4) is too small to support generalizable claims; the results should be interpreted as formative evidence guiding design iteration rather than definitive validation.

To provide robust evidence of practical utility, we plan a larger, randomized multi-site field study across major cotton-growing regions (e.g., southern Xinjiang, Yellow River Basin, and Hubei). The study will use stratified recruitment by region and literacy level, with random assignment to interface variants (e.g., baseline vs. enhanced multi-label narration). Primary outcomes will include task success rate and the System Usability Scale (SUS); secondary outcomes will include voice intelligibility (MOS), error/clarification rates, and time-to-completion. We will preregister the protocol, conduct an *a priori* power analysis (targeting 60–120 participants), and analyze results using mixed-effects models to account for site- and user-level variability. This design directly targets the two critical issues raised by users and the reviewer: multi-label confusion and the need for dialect support and broadcast-speed control.

Nonetheless, this study has several limitations. First, the current training dataset is limited in both size and variety, which may hinder accurate detection of less common or newly emerging cotton diseases and pests. Second, the knowledge graph lacks a real-time incremental update mechanism, so new disease cases or evolving farming practices may not be captured promptly. Third, language support in the voice module is currently restricted, and advanced customization options are not available, which may limit the system’s 857 use among multilingual and diverse user groups.

For future development, it will be important to further expand and diversify the training dataset, strengthen the model’s generalization and its ability to recognize rare diseases, and implement an automated incremental update mechanism for the knowledge graph. On the interaction side, we will further refine multi-label narration via hierarchical summarization, calibrated confidence disclosures, and interactive clarification turns (e.g., “Did you mean A or B?”), while broadening dialect coverage through lexicon expansion and lightweight on-device TTS/ASR adaptation. In addition, extending multi-language capabilities and offering more personalized options in the voice module will be a priority, so the system can better adapt to various agricultural contexts and the needs of different users.

Moreover, soil properties and precise irrigation practices are key factors in the development and management of crop diseases and pests. With advances in Internet of Things (IoT) and intelligent sensing, context-aware smart fertilization and irrigation systems are increasingly used in agriculture. For example, IoT-enabled fertilizer recommendation platforms can monitor soil moisture and nutrient content in real time, adjusting fertilization plans based on crop needs. Intelligent approaches for Reference Evapotranspiration (ETo) allow irrigation to be fine-tuned using climate, soil, and crop water demand data, while context-aware evapotranspiration (ETs) models support more targeted and sustainable irrigation, especially for saline soil remediation. Integrating these IoT-based sensing and reasoning tools with the present system in future work is expected to further improve its capabilities and support the development of intelligent, sustainable agriculture.

## 9 Conclusion

This study developed an intelligent cotton pest and disease recognition and voice interaction system that integrates object detection and knowledge graphs, overcoming the shortcomings of traditional approaches in efficiency, expertise, and timely response. The main contributions are as follows:

1. Introduced the YOLOv11-LDNet model, which uses LAMP pruning and knowledge distillation to achieve efficient lightweight optimization, allowing real-time inference on edge devices even in challenging field settings.
2. Built a comprehensive lifecycle knowledge graph for cotton pests and diseases with Neo4j, covering causes, transmission pathways, and prevention measures to support intelligent Q&A.
3. Applied natural language processing and knowledge graph reasoning for semantic, dynamic Q&A, providing immediate diagnosis and recommendations via voice feedback, with a Q&A accuracy rate above 95%.
4. Deployed the system successfully on the Jetson Xavier NX, utilizing a lightweight design and local reasoning to maintain reliable offline operation.

Overall, the system delivers a closed-loop solution from perception through cognition to action, combining high detection accuracy, real-time operation, and improved user experience.

Future Work: The following directions are planned to further advance this research:

1. We will work to boost real-time performance at scale by exploring advanced model compression methods—such as quantization-aware training and neural architecture search—and by applying adaptive resource management. These approaches are intended to support higher-resolution inputs and multiple simultaneous detection tasks on resource-limited devices.
2. Broader field trials and participatory user studies will be carried out across different agricultural settings to thoroughly evaluate system usability, robustness, and real-world impact. Feedback from these studies will inform iterative improvements in interface design, voice interaction accuracy, and adaptability.
3. The system framework will be extended to detect pests and diseases in other key crops, including wheat, rice, and maize. With input from local agricultural experts, region-specific knowledge graphs will 901 be built to assess the transferability and scalability of the approach.
4. We also plan to integrate multimodal and proactive interaction modules—such as image-based Q&A, mobile advisory services, and weather-linked disease forecasting—to deliver more complete, intelligent 904 support for agricultural decision-making.

These targeted efforts will further reinforce the scientific and practical value of the system and support its broader application in smart agriculture.

## Data Availability

The original contributions presented in the study are included in the article/supplementary material. Further inquiries can be directed to the corresponding author.
